# Utilization of Cardiovascular Procedures, Consultation Services, and Cardioprotective Medications Among Type 2 Myocardial Infarction Patients

**DOI:** 10.1016/j.jacadv.2025.101629

**Published:** 2025-02-20

**Authors:** Harsh Goel, Meera V. Kapadia, Karan V. Goenka, Caroline M. Schaefer, Frances L. Revere, James L. Januzzi, Saumil S. Datar, Cian P. McCarthy, Adwait G. Mehta

**Affiliations:** a Department of Cardiovascular Disease, The University of Texas Health Science Center, Houston McGovern Medical School, Houston, Texas, USA; b Department of Internal Medicine, The University of Texas Health Science Center, Houston McGovern Medical School, Houston, Texas, USA; c Department of Management, Policy and Community Health, The University of Texas Health Science Center, Houston School of Public Health, Houston, Texas, USA; d University of Florida College of Public Health and Health Professionals, Gainesville, Florida, USA; e Division of Cardiology, Department of Medicine, Massachusetts General Hospital, Harvard Medical School, Boston, Massachusetts, USA; f Baim Institute for Clinical Research, Boston, Massachusetts, USA; g Department of Hospital Medicine, The University of Texas MD Anderson Cancer Center, Houston, Texas, USA

**Keywords:** cardioprotective medications, diagnostic testing, revascularization, type 1 myocardial infarction (T1MI), type 2 myocardial infarction (T2MI)

## Abstract

**Background:**

Coronary atherosclerosis and recurrent cardiovascular events are common among individuals with type 2 myocardial infarction (T2MI). However, cardiovascular resource utilization among T2MI patients is unclear.

**Objectives:**

The aim of the study was to characterize cardiovascular resource utilization among T2MI patients across the United States.

**Methods:**

Using Optum’s de-identified Clinformatics Data Mart Database, cardiovascular procedures, physician services, and prescriptions within 6 months postdischarge were compared among patients with T2MI vs type 1 myocardial infarction (T1MI) between October 1, 2017, and June 30, 2020. Multivariable logistic regression examined the odds of resource utilization in T2MI vs T1MI and identified predictors of utilization for T2MI.

**Results:**

We identified 140,344 patients with myocardial infarction; 121,738 patients (87%) had T1MI and 18,606 (13%) had T2MI. All participants had 183 days of postdischarge follow-up. Within 6 months postdischarge, patients with T2MI were significantly less likely to fill new prescriptions for P2Y_12_ inhibitors (4.8% [603/14,176] vs 52.8% [44,833/99,593], adjusted OR: 0.28; 95% CI: 0.25-0.31), beta blockers (27.1% [2,070/14,176] vs 62.8% [38,219/99,593], OR: 0.59; 95% CI: 0.55-0.63), statins (19.1% [1,439/14,176] vs 59.1% [32,434/99,593], OR: 0.51; 95% CI: 0.47-0.55), and SGLT2i or glucagon-like peptide-1 agonists (4.8% [595/14,176] vs 35.4% [30,202/99,593], OR: 0.30; 95% CI: 0.27-0.33) as compared to T1MI. Patients with T2MI were significantly less likely to undergo an echocardiogram (71.8% [10,179/14,176] vs 82.9% [82,551/99,593], OR: 0.61; 95% CI: 0.58-0.64) and coronary angiogram (11.7% [1,664/14,176] vs 76.6% [76,327/99,593], OR: 0.10; 95% CI: 0.09-0.11) compared to T1MI.

**Conclusions:**

In the United States, T2MI patients received less cardiovascular testing and secondary preventative therapies than T1MI patients.

Approximately 3-quarters of a million individuals experience a myocardial infarction (MI) in the United States every year. The pathophysiology of these MIs is diverse, with 5 distinct subtypes outlined in the Universal Definition of MI.^1^ For instance, type 1 MI (T1MI) is characterized by acute plaque rupture or erosion leading to acute thrombus formation. In contrast, type 2 MI (T2MI) is a heterogenous condition caused by a demand/supply mismatch from a variety of potential acute medical illnesses distinctly occurring in the absence of atherothrombosis.^2^ These 2 forms of MI are the most frequently encountered.

It is recognized that T2MI is prevalent, with several studies suggesting this form of MI may be as common, if not more so, than T1MI.^3^^,^^4^ In addition to being common, approximately 1-third of individuals who experience a T2MI have a recurrent major adverse cardiovascular event within 5 years of diagnosis.^5^ Recent prospective studies have revealed that the vast majority of individuals with T2MI have a high prevalence of coronary artery plaque including noncalcified plaque, which may be modifiable.^3^^,^^6^

Despite their high risk of recurrent cardiovascular events and high coronary plaque burden, single-center studies from large academic centers have suggested that individuals with T2MI often do not receive the same amount of diagnostic or therapeutic resources as T1MI.^7^, ^8^, ^9^, ^10^, ^11^ However, it is uncertain if these testing and treatment patterns are consistent across a broad range of health care facilities across the United States. Furthermore, studies to date have predominantly focused on inpatient hospitalization; data regarding resource utilization and medication fills postdischarge is lacking. This is important, as it is plausible that utilization of such resources may have been deferred to the outpatient setting after the patient recovers from their hospitalization.

Given these observations, we sought to characterize utilization patterns of cardiovascular resources in a nationally representative cohort of persons diagnosed with T2MI across the United States. The availability of an International Classification of Disease-10 (ICD-10) diagnosis code for T2MI since October 2017 facilitated such an analysis, which aimed to compare cardiovascular-specific diagnostic and therapeutic procedures, physician services, and cardioprotective medication prescription fill rates among patients with T2MI and T1MI and identify predictive factors for each among T2MI individuals.

## Methods

This study was institutional review boards exempt by review of the University of Texas Health Science Center at Houston, as Optum’s de-identified Clinformatics Data Mart Database (CDM) database is publicly available and contains deidentified patient information.

### Data source

This was a retrospective observational study using CDM. CDM is a large national database that includes approximately 67 million geographically diverse individuals from all 50 states in the United States. Data included in the database are deidentified and managed in accordance with Optum customer data use agreements. Individuals are included in the database by virtue of their commercial or Medicare Advantage insurance coverage. The database captures patient demographics, comorbidities, medical and prescription claims, resource utilization, and clinician information, with claims undergoing verification and adjudication.

### Study population and patient characteristics

ICD-10-Clinical Modification (ICD-10-CM) diagnostic codes were utilized to identify individuals hospitalized with T1MI (121.0, 121.1, 121.2, 121.3, 12.4, 121.9) or T2MI (121.A1) in CDM from October 1, 2017, to June 30, 2020. ICD codes for T1MI, T2MI, and other clinical diagnoses were aggregated using the Clinical Classifications Software Refined. As a component of the Healthcare Cost and Utilization Project, the Clinical Classifications Software Refined groups over 700,000 ICD-10-CM codes into 530+ diagnostic categories (Supplemental Tables 1 to 3). Data included patient demographics, geographic region, comorbidities, index admission diagnosis, cardiovascular testing, procedures, and cardioprotective medication utilization before, during, and within 6 months of the index MI hospitalization. Analysis was limited to individuals with at least 6 months of continuous enrollment before and after hospitalization. Those under the age of 18 years, individuals with Medicare Supplements, or out-of-network claims were excluded.

### Cardioprotective medications

Cumulative fill rates for cardioprotective medications within 6 months of discharge were determined using the standardized American Hospital Formulary Service classification system and the National Drug Code directory. Cardioprotective medications included antiplatelet medications like aspirin or purinergic receptor (P2Y_12_) inhibitors (clopidogrel, prasugrel, ticagrelor), beta-blockers, angiotensin-converting enzyme inhibitors (ACEIs), angiotensin II receptor blockers (ARBs), sacubitril/valsartan (Entresto), cholesterol-lowering medications (statins, ezetimibe, proprotein convertase subtilisin/kexin type 9 [PCSK-9] inhibitors), and diabetes medications such as sodium glucose co-transporter 2 (SGLT-2) inhibitors and glucagon-like peptide-1 (GLP-1) agonists. For each medication class, new prescriptions of each medication within 6 months of hospital discharge were compared to baseline prescription prior to their index MI hospitalization. Importantly, prior use of a specific medication did not preclude a patient from inclusion in analyses for other medication classes for which they had no preadmission prescriptions. This approach ensured that each medication class analysis was restricted to newly initiated treatments postdiagnosis. Nonstandard dosages and combination preparations of aspirin were excluded. Nonstandard formulations of beta-blockers (ie, eyedrops) were excluded.

### Cardiovascular testing, procedures, and physician services

The ICD-10 Revision Procedure Coding System (ICD-10-PCS), Current Procedural Terminology codes, Healthcare Common Procedure Coding System (Supplemental Table 4), and CDM protocols were utilized to identify cardiovascular diagnostic and therapeutic procedures (collectively known as procedures) performed in-hospital and up to 6 months postdischarge, as well as cardiovascular specialty consultations during the initial admission and follow-up visits (diagnostic cardiovascular procedures included 2-dimensional echocardiogram complete, echocardiogram stress test, exercise stress test, nuclear stress test, diagnostic coronary angiogram, and computed tomography coronary angiogram). Therapeutic cardiovascular procedures encompassed coronary angiograms with therapeutic or interventional procedures (percutaneous coronary intervention [PCI]) and coronary artery bypass graft (CABG). Additionally, primary care physician (PCP) visits up to 6 months postdischarge were recorded.

### Statistical analysis

Comparative analyses of patients with a primary or secondary diagnosis of isolated T1MI vs those with a primary or secondary diagnosis of isolated T2MI were conducted. Patients with a dual index diagnosis of T1MI and T2MI were excluded as the primary goal of our analyses was to understand how resource utilization differs for these 2 subtypes of MI. A sensitivity analysis was performed comparing the characteristics of individuals with T2MI and those with a dual index diagnosis of T1MI and T2MI, which revealed their characteristics to be similar (Supplemental Table 5). Comparative analyses examined demographics, geographic region, comorbidities, index admission diagnoses arranged in groups, cardioprotective medication use before (defined as prescription fills within 6 months prior to hospitalization) and during index admission or up to 6 months after hospitalization, as well as cardiovascular procedures before (defined as procedures performed within 6 months of hospitalization) and during index admission or up to 6 months after hospitalization. Comparison was done regarding the utilization of cardiovascular physician services during index admission and PCP/cardiovascular visits up to 6 months postdischarge. Continuous variables were analyzed using Student’s t-test and categorical variables with the chi-square or Fisher’s exact test.

Regression analysis utilizing multivariable logistic regression was used to determine unadjusted and adjusted ORs for new cardioprotective medication prescription, cardiovascular procedures, and cardiology or PCP service utilization in patients with isolated T2MI vs isolated T1MI. Utilization was defined as at least 1 new medication fill, procedure or physician encounter during the index admission or up to 6 months postdischarge from the hospital. Medication prescription ORs were calculated as the odds of new medication prescriptions in T2MI divided by the odds of new medication prescriptions in T1MI, with an OR <1 indicating lower odds of prescription fills. Similarly, OR for cardiovascular procedures and consultation were calculated as the odds of new procedures or consultations in T2MI divided by the odds of new procedures or consultations in T1MI, with an OR <1 indicating lower odds of procedures or consultation. For this analysis, patients were excluded who: 1) died during the index hospitalization; 2) discharged to hospice services; or 3) had a dual diagnosis of T2MI and T1MI.

For the regression analysis, 2 separate models with different covariates and outcomes were used. In the first model, assessing the OR of cardioprotective medication fills, control variables included demographics (gender, age, and race), geographic region, comorbidities, admission diagnostic group, index and postdischarge (within 6 months) cardiac procedures, index cardiology consults, and postdischarge cardiology and PCP follow-ups (within 6 months of discharge). In the second model, evaluating the OR of cardiovascular diagnostic tests, revascularization procedures, and cardiovascular physician evaluations, control variables included demographics (gender, age, race), geographic region, comorbidities, admission diagnostic group, preadmission cardiac diagnostic and therapeutic procedures, index cardiology consults, and postdischarge cardiology and PCP follow-ups (within 6 months of discharge). In both models, cardiac procedures were grouped into 3 categories: coronary artery disease (CAD) specific (echocardiogram stress test, exercise stress test, nuclear stress test, angiogram, and computed tomography coronary angiogram), non-CAD specific (echocardiogram), and therapeutic (PCI and CABG). The analyses were conducted using SAS 9.4.

## Results

### Patient characteristics

There were 140,344 individuals hospitalized with an isolated T1MI or T2MI over the 33-month study period: 121,738 (87%) had T1MI alone and 18,606 (13%) had T2MI alone (Table 1). All participants had 183 days postdischarge follow-up.

**Table 1:** Baseline Characteristics of Patients With Myocardial Infarction

	MI Type		*P* Value
	T1MI (n = 121,738)	T2MI (n = 18,606)	
Age, y			**<0.001**
Under 65	27,993 (22.99)	2,638 (14.18)	
65-69	17,422 (14.31)	2,087 (11.22)	
70-74	20,471 (16.82)	2,751 (14.79)	
75-79	19,185 (15.76)	3,030 (16.29)	
80-84	15,478 (12.71)	2,853 (15.33)	
85+	21,189 (17.41)	5,247 (28.2)	
Mean ± SD	72.25 ± 11.82	75.94 ± 11.38	
Sex			**<0.001**
Female	50,640 (41.6)	9,346 (50.23)	
Male	71,098 (58.4)	9,260 (49.77)	
Race			**<0.001**
Asian	3,122 (2.56)	455 (2.45)	
Black	16,082 (13.21)	2,980 (16.02)	
Hispanic	13,404 (11.01)	1,710 (9.19)	
White	89,130 (73.21)	13,461 (72.35)	
U.S. region			**<0.001**
Midwest	26,754 (21.98)	4,015 (21.58)	
Northeast	15,641 (12.85)	3,779 (20.31)	
South	56,991 (46.81)	7,190 (38.64)	
West	22,352 (18.36)	3,622 (19.47)	
Charlson comorbidity index	4 (2, 6)	5 (3, 7)	<0.001
Comorbidities^a^			
Atrial fibrillation, flutter, PSVT, VT	47,915 (39.36)	10,370 (55.73)	<0.001
Chronic ischemic heart disease	106,164 (87.21)	10,775 (57.91)	<0.001
Hyperlipidemia	104,462 (85.81)	14,671 (78.85)	<0.001
Smoking	31,799 (26.12)	4,042 (21.72)	<0.001
Peripheral vascular disease	22,321 (18.34)	3,920 (21.07)	<0.001
CVA, TIA, carotid stenosis, intracranial atherosclerotic disease	36,010 (29.58)	6,488 (34.87)	<0.001
Pulmonary embolism/venous embolism or thrombosis	9,940 (8.17)	2,870 (15.43)	<0.001
Diabetes (1 and 2)	60,848 (49.98)	9,549 (51.32)	0.001
COPD and asthma	42,498 (34.91)	8,716 (46.85)	<0.001
Anemia and other coagulation disorders	42,802 (35.16)	9,724 (52.26)	<0.001
CHF and exacerbation	62,228 (51.12)	12,005 (64.52)	<0.001
Liver diseases	14,085 (11.57)	3,340 (17.95)	<0.001
Kidney diseases	59,196 (48.63)	13,028 (70.02)	<0.001
Hypertension and crisis	112,097 (92.08)	17,484 (93.97	<0.001
Gastrointestinal diseases	12,038 (9.89)	3,247 (17.45)	<0.001

Values are n (%), mean ± SD, or median (Q1,Q3).

CVA = cerebrovascular accident; CHF = congestive heart failure; COPD = chronic obstructive pulmonary disease; MI = myocardial infarction; PSVT = paroxysmal supraventricular tachycardia; TIA = transient ischemic attack; T1MI = type 1 MI; T2MI = type 2 MI; VT = ventricular tachycardia.

a Present within 6 months of index admission.

Individuals with isolated T2MI were older (mean age 75.9 ± 11.4 years vs 72.3 ± 11. years), more commonly female (50.2% [9,346/18,606] vs 41.6% [50,640/121,738]), and more likely to be Black (16.0% [2,980/18,606] vs 13.2% [16,082/121,738]) than those with isolated T1MI.

Individuals with T2MI had a higher burden of comorbidities than those with T1MI as suggested by their Charlson comorbidity index (median weighted index of 5 [IQR: 3-7] vs 4 [IQR: 3-7]). Individuals with T2MI had a higher prevalence of prior arrhythmias (55.7% [10,370/18,606] vs 39.4% [47,915/121,738]), heart failure (64.5% [12,005/18,606] vs 51.1% [62,228/121,738]), kidney disease (70.0% [13,028/18,606] vs 48.6% [59,196/121,738]), prior cerebrovascular disease (34.9% [6,488/18,606] vs 29.6% [36,010/121,738]), than persons with T1M1. Conversely, certain atherosclerotic risk factors such as smoking (26.1% [31,799/121,738] vs 21.7% [4,042/18,606]), hyperlipidemia (85.8% [104,462/121,738] vs 78.9% [14,671/18,606]), and established chronic ischemic heart disease (87.2% [106,164/121,738] vs 57.9% [10,775/18,606]) were more common in individuals with T1MI than those with T2MI (Table 1). Individuals with T2MI were more likely to be admitted to a medical or surgical floor and less likely to be admitted to a cardiology floor than individuals with T1MI (Supplemental Table 6).

Cardioprotective medications prior to admission, while differing by statistical significance due to the large sample size, were numerically relatively similar among individuals with T2MI and T1MI including statins (45.1% [8,397/18,606] vs 43.8% [53,323/121,738], *P* < 0.001), ACEI/ARB (41.5% [7,727/18,606] vs 42.8% [52,048/121,738], *P* = 0.002), and beta blockers (44.6% [8,291, 18,606] vs 38.5% [46,860/121,738] *P* < 0.001), respectively (Supplemental Table 7).

Cardiovascular procedures prior to admission also differed by statistical significance but were numerically similar for most tests. However, a greater number of individuals with T2MI underwent an echocardiogram within 6 months of admission (41.1% [7,637/18,606] vs 28.4% [34,562/121,738]) (Supplemental Table 7).

### Primary hospital diagnosis by MI subtype

Infectious diseases, diseases of the cardiovascular system, and respiratory system were the most common diagnostic groups among individuals with T2MI. In contrast, disorders of the cardiovascular system were the most common primary diagnostic group among individuals with T1MI (Table 2).

**Table 2:** Primary Hospital Admission Diagnosis by MI Subtype

	MI Subtype		*P* Value
	T1MI (n = 121,738)	T2MI (n = 18,606)	
Primary diagnosis			**<0.001**
Disorders of the blood and blood-forming organs and disorders involving the immune mechanism	210 (0.17)	177 (0.95)	
Disorders of the cardiovascular system	97,879 (80.4)	6,080 (32.68)	
Disorders of the gastrointestinal system	1,901 (1.56)	1,048 (5.63)	
Diabetes and disorders of the endocrine system, nutrition, and metabolism	659 (0.54)	278 (1.49)	
Disorders of the genitourinary system	1,122 (0.92)	730 (3.92)	
Infectious diseases	11,083 (9.1)	6,494 (34.9)	
Disorders of the musculoskeletal system and injuries	1,735 (1.43)	738 (3.97)	
Neoplastic diseases	733 (0.6)	392 (2.11)	
Disorders of the neurological and cerebrovascular system	928 (0.76)	452 (2.43)	
Disorders of perioperative care	2,245 (1.84)	503 (2.7)	
Disorders of the respiratory system	2,176 (1.79)	1,210 (6.5)	
Other	1,047 (0.86)	495 (2.66)	

Values are n (%).

Abbreviations as in Table 1.

### Diagnostic testing and cardiology evaluation according to MI subtype

**Figure 1:**
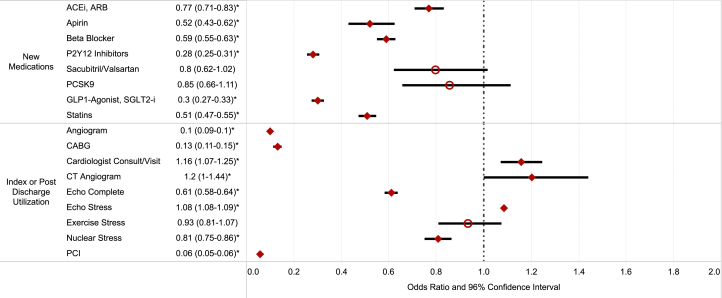
Multivariable Logistic Regression Comparing Odds of Cardiovascular Medications, Procedures, and Consultations for T2MI vs T1MI Utilizing multivariable logistic regression, unadjusted and adjusted OR were determined for new cardioprotective medication prescriptions, procedures, and cardiology or PCP services utilization in patients with isolated T2MI vs T1MI within 6 months of the index hospitalization. ACEI = angiotensin-converting enzyme inhibitor; ARB = angiotensin receptor blocker; CABG = coronary artery bypass graft; CT = computed tomography; ECHO = echocardiogram; GLP = glucagon-like peptide-1; MI = myocardial infarction; P2Y12 = purinergic receptor inhibitor; PCI = percutaneous coronary intervention; PCP = primary care physician; PCSK9 = paraprotein convertase subtilisin/kexin type 9; red circle = statistically insignificant; red diamond = statistically significant; SGLT2-i = sodium glucose co-transporter 2 inhibitor; T1MI = type 1 MI; T2MI = type 2 MI.

Individuals with T2MI underwent fewer diagnostic tests during their index hospitalization and within 6 months postdischarge compared to T1MI (Table 3, Figure 1). This included diagnostic echocardiography (71.8% [10,179/14,176] vs 82.9% [82,551/99,593], adjusted OR: 0.61; 95% CI: 0.58-0.64) and invasive coronary angiogram (11.7% [1,664/14,176] vs 76.6% [76,327/99,593], adjusted OR: 0.1; 95% CI: 0.09-0.11).

**Table 3:** New Diagnostic Test, Therapeutic Procedure, or Cardiology Consultation According to MI Subtype

Procedures During Index Admit or Within 6 Mo of Discharge	T1MI (n^a^ = 99,593)	T2MI (n^a^ = 14,176)	Odds of Procedure/Intervention in T2MI vs T1MI	
			Unadjusted	Adjusted^b^
Echo complete	82,551 (82.89)	10,179 (71.80)	0.53 (0.51-0.55)	0.61 (0.58-0.64)
Echo stress	477 (0.48)	47 (0.33)	0.69 (0.51-0.93)	0.92 (0.67-1.25)
Stress exercise	3,245 (3.26)	275 (1.94)	0.59 (0.52-0.67)	0.93 (0.81-1.07)
Stress nuclear	9,388 (9.43)	1,253 (8.84)	0.93 (0.88-0.99)	0.81 (0.75-0.86)
Angiogram	76,327 (76.64)	1,664 (11.74)	0.04 (0.04-0.04)	0.10 (0.09-0.10)
CT angiogram	1,132 (1.14)	172 (1.21)	1.07 (0.91-1.26)	1.20 (1.00-1.44)
PCI	49,470 (49.67)	258 (1.82)	0.02 (0.02-0.02)	0.06 (0.05-0.06)
CABG	18,292 (18.37)	210 (1.48)	0.07 (0.06-0.08)	0.13 (0.11-0.15)
Cardiology consult	86,275 (86.63)	12,913 (91.09)	1.58 (1.49-1.68)	1.16 (1.07-1.25)

Values are n (%) or OR (95% CI). New diagnostic or therapeutic procedure or cardiology evaluation up to 6 months postdischarge from index MI admission.

CABG = coronary artery bypass graft; CT = computed tomography; ECHO = echocardiogram; PCI = percutaneous coronary intervention; other abbreviations as in Table 1.

a Patients who expired in hospital, were discharged to hospice, or did not have 6 months of continuous enrollment were excluded.

b Adjusted for all demographics, comorbidities, primary diagnostic group, cardiac procedures, and physician evaluations.

Conversely, patients with T2MI were more frequently evaluated by a cardiologist during hospitalization or as outpatients up to 6 months postdischarge (91.1% [12,913/14,176] vs 86.6% [86,275/99,593], adjusted OR: 1.16; 95% CI: 1.07-1.25) as compared to patients with T1MI. They were also slightly more likely to undergo a computed tomography coronary angiogram (1.2% [172/14,176] vs 1.1% [1,132/99,593], adjusted OR: 1.20; 95% CI: 1.07-1.25) (Table 3, Figure 1).

In a sensitivity analysis comparing T1MI to primary diagnosis T2MI (Supplemental Table 8), individuals with primary diagnosis T2MI were still less likely to undergo an echocardiogram (75.0% [481/641] vs 82.9% [82,551/99,593], adjusted OR: 0.55; 95% CI: 0.46-0.67) and an invasive angiogram (28.2% [181/641] vs 76.6% [76,327/99,593], adjusted OR: 0.12; 95% CI: 0.09-0.14) than those with T1MI. However, they were more likely to undergo a nuclear stress test (17.0% [109/461] vs 9.4% [9,388/99,593], adjusted OR: 1.56; 95% CI: 1.26-1.93).

### Invasive treatment of MI according to MI subtype

A substantially lower number of individuals with T2MI underwent PCI (1.8% [258/14,176] vs 49.7% [49,470/99,593], adjusted OR: 0.06; 95% CI: 0.05-0.06) and CABG (1.5% [210/14,176] vs 18.4% [18,292/99,593], adjusted OR: 0.13; 95% CI: 0.11-0.15) during index admission and within 6 months postdischarge as compared to T1MI (Table 3, Figure 1).

The portion of individuals undergoing revascularization (PCI or CABG) following an initial angiogram (computed tomography coronary angiogram or invasive angiogram) was significantly higher in T1MI patients compared to T2MI patients (79.3% [60,596/76,385] vs 23.8% [414/1,740] *P* < 0.001) (Supplemental Table 9).

In a sensitivity analysis, comparing T1MI to primary diagnosis T2MI (Supplemental Table 8) individuals with T2MI, individuals with primary diagnosis T2MI were also less likely to undergo PCI (5.9% [38/641] vs 49.7% [49,470/99,593], adjusted OR: 0.08; 95% CI: 0.06-0.12) and CABG (5.0% [32/641] vs 18.4% [18,292/99,593], adjusted OR: 0.25; 95% CI: 0.17-0.37) than persons with T1MI.

### Cardioprotective medication utilization according to MI subtype

Postdischarge, T2MI patients had significantly lower fill rates for secondary preventive therapies compared to T1MI patients across all major cardioprotective drug categories (Table 4, Figure 1), among those naïve to these medications at admission (ie, no prescription fill 6 months prior to MI hospitalization).

**Table 4:** New Secondary Prevention Prescriptions According to MI Subtype

	T1MI (n^a^ = 99,593)	T2MI (n^a^ = 14,176)	Odds of New Prescription in T2MI vs T1MI	
			Unadjusted	Adjusted^b^
ACEI or ARB naive	55,667 (55.89)	7,952 (56.09)		
New prescription	19,036 (34.20)	1,030 (12.95)	0.29 (0.27-0.31)	0.77 (0.71-0.83)
Aspirin naive	98,345 (98.75)	14,040 (99.04)		
New prescription	7,566 (7.69)	158 (1.13)	0.14 (0.12-0.16)	0.52 (0.43-0.62)
Beta-blocker naive	60,817 (61.07)	7,630 (53.82)		
New prescription	38,219 (62.84)	2,070 (27.13)	0.22 (0.21-0.23)	0.59 (0.55-0.63)
P2Y_12_ inhibitor naïve	84,998 (85.35)	12,515 (88.28)		
New prescription	44,833 (52.75)	603 (4.82)	0.05 (0.04-0.05)	0.28 (0.25-0.31)
Sacubitril/valsartan naive	97,390 (97.79)	13,939 (98.33)		
New prescription	1,212 (1.24)	90 (0.65)	0.52 (0.42-0.64)	0.80 (0.62-1.02)
PCSK9i or ezetimibe naive	97,534 (97.93)	13,956 (98.45)		
New prescription	2,269 (2.33)	74 (0.53)	0.22 (0.18-0.28)	0.85 (0.66-1.11)
GLP1-agonist or SGLT2-i naive	85,341 (85.69)	12,418 (87.60)		
New prescription	30,202 (35.39)	595 (4.79)	0.09 (0.08-0.10)	0.30 (0.27-0.33)
Statin naive	54,913 (55.14)	7,554 (53.29)		
New prescription	32,434 (59.06)	1,439 (19.05)	0.16 (0.15-0.17)	0.51 (0.47-0.55)

Values are n (%) or OR (95% CI). New prescriptions up to 6 months postdischarge from index MI admission.

ACEI = angiotensin converting enzyme inhibitor; ARB = angiotensin receptor blocker; GLP1 = glucagon-like peptide-1; P2Y12i = purigenic receptor inhibitor; PCSK9i = paraprotein convertase subtilisin/kexin type 9 inhibitor; SGLT2-i = sodium glucose co-transporter 2 inhibitor.

a Patients who expired in hospital, discharged to hospice or did not have 6 months of continuous enrollment were excluded.

b Adjusted for all demographics, comorbidities, primary diagnostic group, cardiac procedures, and physician evaluations.

Among medication naïve individuals at the time of the index event, within 6 months of their hospitalization, individuals with T2MI were significant less likely to fill a new prescription for ACEI/ARBs (13.0% [1,030/7,952] vs 34.2% [19,06/55,667], adjusted OR: 0.77; 95% CI: 0.71-0.83), statins (19.1% [1,439/7,554] vs 59.1% [32,434/54,913], adjusted OR: 0.51; 95% CI: 0.47-0.55), P2Y_12_ inhibitors (4.8% [603/12,515] vs 52.8% [44,933/84,998], adjusted OR: 0.28; 95% CI: 0.25-0.31), beta blockers (27.1% [2,070/7,630] vs 62.8% [38,219/60,817], adjusted OR: 0.59; 95% CI: 0.55-0.63), nonstatin lipid-lowering medications such as PCSK9i or ezetimibe (0.5% [74/13,956] vs 2.3% [2,269/97,534], adjusted OR: 0.85; 95% CI: 0.66-1.11) or SGLT2i or GLP-1 agonists (4.8% [595/12,418] vs 35.4% [30,202/85,341], adjusted OR: 0.3; 95% CI: 0.27-0.33) as compared to individuals with T1MI, respectively (Table 4, Figure 1).

### Predictors of new cardiovascular procedure and consultation following T2MI

**Figure 2:**
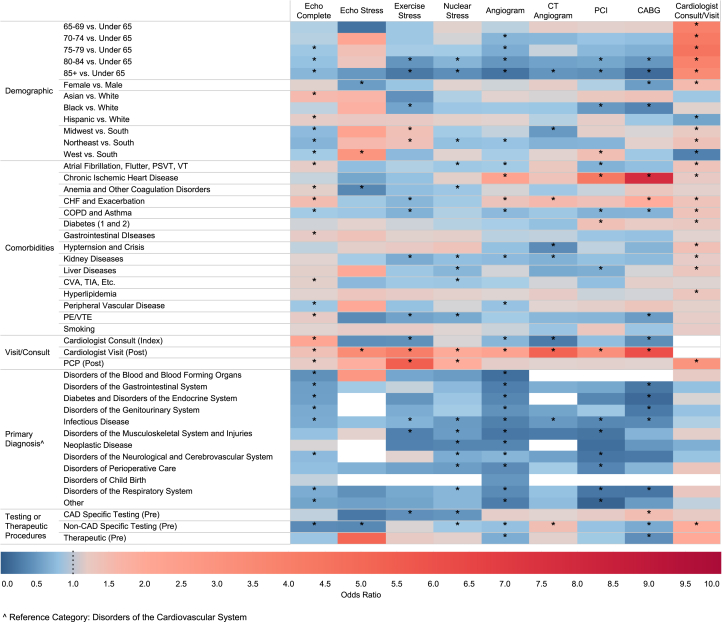
Predictors of Cardiovascular Procedures and Consultations Among T2MI Patients Heat map showing individual variables' effect on procedure or physician service utilization: blue (OR <1), red (OR >1), darker shades (OR further from 1). ∗Statistically significant. White boxes indicate covariates with null values. Cardiac procedures were grouped into 3 categories: CAD specific (echocardiogram stress test, exercise stress test, nuclear stress test, angiogram, and computed tomography coronary angiogram), non-CAD specific (echocardiogram), and therapeutic (PCI and CABG). CAD = coronary artery disease; CHF = congestive heart failure; COPD = chronic obstructive pulmonary disease; CVA = cerebrovascular accident; PE = pulmonary embolism; Pre = pre-hospitalization; PSVT = paroxysmal supraventricular tachycardia; TIA = transient ischemic attack; VT = ventricular tachycardia; VTE = venous thromboembolism; other abbreviations as in Figure 1.

Predictors of new cardiovascular procedures or consultations following T2MI are illustrated in (Figure 2, Supplemental Figures 1 to 9). Advancing age was strongly associated with utilization of cardiology consultations. In contrast, advancing age was associated with reduced utilization of diagnostic and therapeutic procedures. Females were more likely to receive postdischarge cardiology consultations but less likely to undergo stress echocardiogram and CABG. Racial disparities were also noted with Black individuals with T2MI having lower odds of undergoing exercise tolerance test, PCI, and CABG compared to White individuals. Compared to White individuals, Hispanic and Asian individuals were more likely to have an echocardiogram, while a Hispanic patient was less likely to have a cardiology consultation during admission or as an outpatient.

Similar to medication fill rates, comorbidities had varying levels of influence on the likelihood of procedures and consultation (Figure 2, Supplemental Figures 1 to 9). Patients with heart failure were more likely to be evaluated with diagnostic echocardiography. In addition, patients with heart failure and chronic ischemic heart disease were more likely to undergo invasive coronary angiogram, PCI, and CABG. Conversely, patients with kidney disease and chronic obstructive lung disease or asthma were less likely to undergo procedures.

Admission and postdischarge follow-up significantly impacted procedure likelihood. An index cardiology consultation was associated with a high odd of echocardiogram but a lower likelihood of exercise stress test, invasive coronary angiogram, and CABG. Postdischarge cardiologist visit was associated with higher odds of all examined procedures. Similarly, a postdischarge primary care provider visit was linked to higher odds of echocardiogram, exercise stress test, nuclear stress, and cardiology consultation.

Primary diagnosis during admission also played a role. Patients admitted with diagnoses other than cardiovascular disorders had lower odds of undergoing procedures and consultation. To varying degrees, preadmission CAD-specific and non-CAD-specific therapeutic procedures were associated with a statistically significant reduction in utilization of procedures.

### Predictors of new cardiovascular medication prescription following T2MI

**Figure 3:**
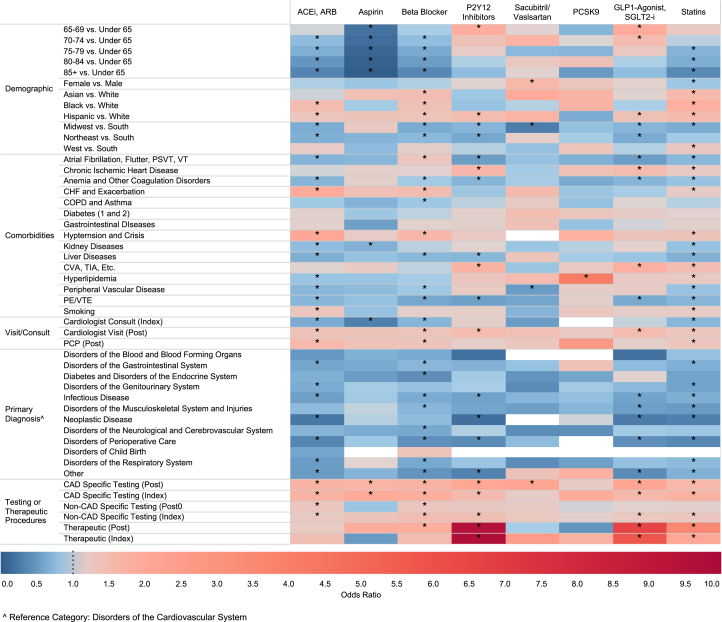
Predictors of Cardiovascular Medication Utilization Among T2MI Patients Heat map showing individual variables' effect on new cardioprotective medication prescription fill: blue (OR < 1), red (OR > 1), darker shades (OR further from 1). ∗Statistically significant. White boxes indicate covariates with null values. Cardiac procedures were grouped into three categories: CAD specific (echocardiogram stress test, exercise stress test, nuclear stress test, angiogram, and computed tomography coronary angiogram), non-CAD specific (echocardiogram), and therapeutic (PCI and CABG). ETT = exercise tolerance/stress test; Index = index hospitalization; P2Y12 = purigenic receptor inhibitor; PCI = percutaneous coronary intervention; PCSK9 = paraprotein convertase subtilisin/kexin type 9; PE = pulmonary embolism; Post = postdischarge; PSVT = paroxysmal supraventricular tachycardia; other abbreviations as in Figures 1 and 2.

Predictors of new cardiovascular prescription fill for each individual medication group are illustrated in Figure 3 and Supplemental Figures 10 to 17 for individuals with T2MI. Advancing age was a predictor for lack of new prescriptions of aspirin, statins, beta blockers, and ACEI/ARB prescription, but advanced age did not predict the odds of a new prescription of P2Y_12_ inhibitor, adjunct lipid-lowering therapy, or sacubitril/valsartan. Females were less likely to be prescribed statins. Black individuals with T2MI were more likely than White individuals to receive a new prescription for an ACEI/ARB, beta blocker, and statin following their index event. Compared to White individuals, Hispanic individuals with T2MI were more likely to receive a new prescription for an ACEI/ARB, beta-blocker, P2Y12i, GLP1 agonist, SGLT2i, and statins.

Comorbidities had varying effects on the odds of prescription fill according to the class of medication (Figure 3, Supplemental Figures 10 to 17). For instance, individuals with heart failure were more likely to be prescribed an ACEI/ARB, beta blocker, and statin. A postdischarge PCP or cardiologist visit was associated with higher odds of a new ACEI/ARB, beta blocker, and statin medication fill within 6 months of the T2MI event. Having an admission diagnosis other than a disorder of the cardiovascular system was associated with a lower likelihood of being prescribed a new cardiovascular medication.

Diagnostic testing and therapeutic interventions, both during the index admission and postdischarge, were generally associated with new medication prescriptions, demonstrating varying positive ORs and levels of statistical significance.

## Discussion

In this national study of individuals with T1MI and T2MI in the United States, significant differences in cardiovascular resource utilization were observed within 6 months of the index event (Central Illustration), aligning with findings from smaller, single-center studies but adding important information focused on the postdischarge period to 6 months following the event. Individuals with T2MI were significantly less likely to receive new prescriptions for cardioprotective therapies following their MI. Additionally, T2MI patients were less likely than T1MI patients to undergo cardiac testing and revascularization procedures during both the index hospitalization and within 6 months postdischarge. Finally, in an effort to understand these gaps, we describe predictors of new cardiovascular prescriptions, procedures, and consultations among individuals with T2MI.

**Central Illustration:**
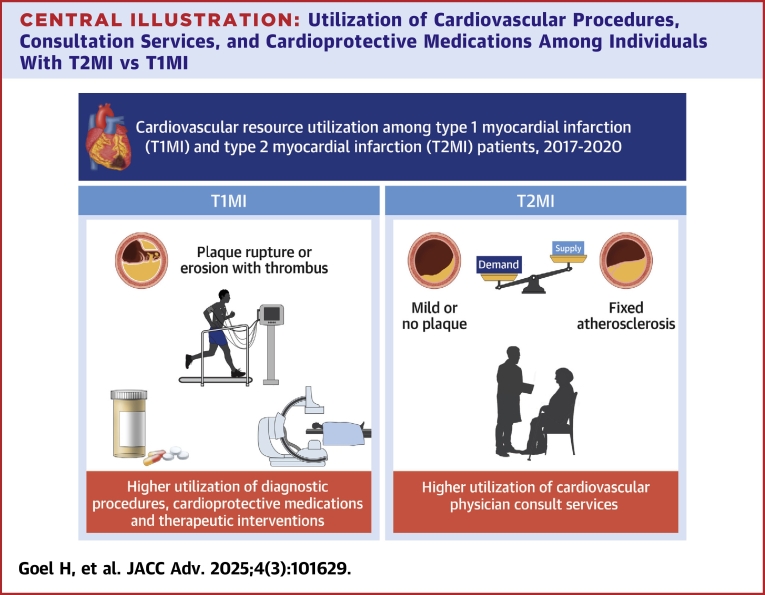
Utilization of Cardiovascular Procedures, Consultation Services, and Cardioprotective Medications Among Individuals With T2MI vs T1MI Figure illustrating key differences in evaluation and treatment of these subtypes of MI. Abbreviations as in Figure 1.

Although noncardiovascular mortality is higher among individuals with T2MI than among those with T1MI, recurrent cardiovascular event rates are not inconsequential with 1-third of individuals experiencing a recurrent MI, stroke, or cardiovascular death over 5 years.^5^ Indeed, crude recurrent cardiovascular event rates for T2MI are comparable to T1MI.^5^^,^^12^^,^^13^ Accordingly, efforts to mitigate cardiovascular risk following a T2MI event are needed. Recent studies indicate a high burden of coronary artery disease and heart failure among individuals with T2MI suggesting a potential role for investigations and therapies to mitigate cardiovascular risk.^6^^,^^14^ For instance, the prospective DEMAND-MI (Determining the Mechanism of Myocardial Injury and Role of Coronary Disease in Type 2 Myocardial Infarction) study observed that 68% of consecutively enrolled T2MI patients had coronary artery disease with obstructive disease identified in 30% by invasive coronary angiography.^6^ Similarly, the DEFINE-TYPE 2 MI (DEFINing the prEvalence and characteristics of coronary artery disease among patients with TYPE 2 Myocardial Infarction using CT-FFR) study detected coronary plaque in 92% of individuals with T2MI by coronary computed tomographic angiography.^3^ The most recent American College of Cardiology/American Heart Association MI guidelines do not segregate treatment for non-ST segment elevation MI according to the subtype of MI (T1MI vs T2MI).^15^ However, small observational studies predominantly from single hospital systems have noted low utilization of secondary preventative therapies among individuals with T2MI at the time of hospital discharge.^5^^,^^9^^,^^10^^,^^16^^,^^17^

The ICD-10 code for T2MI created an opportunity to extend findings from single-center studies, which were typically small and usually only included results to the time of hospital discharge. This analysis of a large U.S. cohort shows that T2MI patients are significantly less likely to receive cardiovascular diagnostic and therapeutic resources than T1MI patients, both during hospitalization and up to 6 months postdischarge, a timepoint at which functional recovery from the hospitalization would have been expected to have occurred. Accordingly, opportunities to increase resource utilization for T2MI patients and implementation trials could clarify the benefits. To our knowledge, these data are among the first to evaluate postdischarge patterns of care in T2MI, an increasingly prevalent and highly morbid diagnosis.

Noteworthy findings in this study include the observation that cardiovascular consultation services were utilized more frequently in T2MI compared to T1MI, highlighting the diagnostic challenges in distinguishing between the 2.^18^ The discrepancy may be attributed to specific patient care and the corresponding role of cardiovascular physicians, either as “primary” providers or in more consultative roles. We observed that individuals with T2MI were more likely to be admitted to a medicine or surgical ward and less likely to a cardiology unit as compared to T1MI. An initial cardiology evaluation was associated with lower utilization of cardioprotective medications such as ACEI/ARBs, aspirin, beta-blockers, and statins, perhaps due to the lack of standardized guidelines. However, postdischarge diagnostic and therapeutic procedures were more common in patients who had a cardiology evaluation after discharge. Advancing age reduced the likelihood that an individual with a T2MI would be started on secondary preventative therapies. Given that individuals with T2MI tend to be older than those with T1MI and also have more medical comorbidities, understanding the cause and approach to manage this observation is important; further studies examining the net benefit of secondary preventative therapies for T2MI in this older population are needed. Furthermore, in this study, individuals with T2MI who underwent cardiovascular testing were more likely to be initiated on secondary preventative therapies. This raises the hypothesis that performing a coronary and structural heart disease evaluation in this population may result in escalation in secondary preventative treatment. Indeed, in the DEMAND MI study, coronary artery disease and structural heart disease were frequently newly discovered and often led to a change in medical therapy.^6^ Whether this testing and treatment initiation improves outcomes, however, remains uncertain. Clinical trials are being initiated to better understand the impact of noninvasive cardiovascular testing on T2MI patient outcomes (NCT05419583). Similar to prior studies,^19^^,^^20^ very low rates of inpatient invasive coronary angiography and revascularization occurred among individuals with T2MI in this study; extending previous work, this study also found very few patients undergo invasive angiography and revascularization after discharge and hospital recovery. Although it is important to note that approximately 24% (414/1,740) of patients with T2MI who underwent angiography subsequently received revascularization therapy, either through PCI or CABG. These data highlight the uncertainty regarding the role of revascularization for T2MI. An ongoing clinical trial is exploring the role of early invasive coronary angiography among individuals with T2MI.^21^

This study has several unique strengths. Firstly, this large, nationally representative dataset encompasses a diverse range of health care facilities, providing comprehensive insights. Secondly, several key predictors influencing medication fills in T2MI patients were identified by meticulously analyzing variables such as patient demographics, comorbidities, and primary diagnoses, offering a precise reflection of current clinical decision-making in T2MI management. Lastly, by using insurance claims data, actual patient-specific pharmaceutical filling patterns were captured, an important strength as nonadherence to cardiovascular medications is common.^22^

Despite its strengths, this study is subject to several limitations that merit consideration. First, ICD coding was used to identify MI events, and thus, misdiagnosis of T1MI or T2MI is a possibility. Limited studies to date have suggested higher agreement for T1MI ICD-10 codes than T2MI codes and adjudicated events using the 4th Universal definition of MI.^23^ Although claims data demonstrates modest agreement with adjudicated cases of MI,^24^ less is known regarding the accuracy of MI ICD-10 coding specifically in CDM. Second, prescriptions were identified via pharmacy claims; however, it is possible that patients may obtain medications over-the-counter. For instance, the rates of aspirin prescriptions were substantially lower than anticipated despite exploring various dataset permutations using American Hospital Formulary Service and National Drug Codes; this may be due to over-the-counter use of the drug. Indeed, a prior survey of patients in the United States revealed that 84% of patients receive aspirin over-the-counter rather than through their insurance.^24^ Third, while measuring prescription fills are a well-accepted metric of medication use and adherence,^25^ they have limitations. Patients may not take their medications exactly as prescribed, and the lack of medical record access precluded ability to assess health care providers' intentions regarding prescriptions, which are typically indicated in discharge summaries or discharge orders. Thus, discharging providers may have intended to treat patients with prescribed medications that were not filled by patients for various reasons. Lastly, as the database primarily includes commercially insured individuals with regional variations, the results may not necessarily generalize to individuals of lower socioeconomic status or certain geographical regions.

## Conclusions

Despite a high expected prevalence of coronary artery disease and elevated rates of recurrent cardiovascular events, patients with T2MI were significantly less likely to be prescribed cardioprotective medications, undergo cardiovascular testing, and undergo revascularization when compared to those with T1MI. This discrepancy underscores the urgent need for clinical trials to identify optimal management strategies for T2MI, including whether enhanced cardiovascular resource utilization can improve outcomes for affected individuals.

## Funding support and author disclosures

Dr Revere provided institutional support for data access and expertise. Dr Januzzi is supported in part by the Adolph Hutter Professorship at Harvard Medical School. Dr McCarthy is supported by a 10.13039/100000050National Heart, Lung, and Blood Institute Career Development Award (K23HL167659). Dr Januzzi reports a board position with Imbria Pharma, grant support from Abbott, Applied Therapeutics, AstraZeneca, BMS, Novartis Pharmaceuticals, consulting income from Abbott Diagnostics, Beckman-Coulter, Jana Care, Janssen, Novartis, Prevencio, Quidel, and Roche Diagnostics and serves on clinical endpoint committees/data safety monitoring boards for Abbott, AbbVie, Amgen, CVRx, Medtronic, Pfizer, and Roche Diagnostics. Dr McCarthy has received consulting fees/honorarium from Abbott Laboratories, Roche Diagnostics, HeartFlow, Inc, and New Amsterdam Pharma. All other authors have reported that they have no relationships relevant to the contents of this paper to disclose.

Perspectives **COMPETENCY IN PRACTICE BASED LEARNING:** Despite a high expected prevalence of coronary artery disease and elevated rates of recurrent cardiovascular events, patients with T2MI were significantly less likely to be prescribed cardioprotective medications or undergo cardiovascular testing and revascularization when compared to those with T1MI.

**TRANSLATIONAL OUTLOOK:** This discrepancy underscores the urgent need for clinical trials to identify optimal management strategies for T2MI, including whether enhanced cardiovascular resource utilization can improve outcomes for affected individuals.
