# Supplementary material for: Utilization of Cardiovascular Procedures, Consultation Services, and Cardioprotective Medications Among Type 2 Myocardial Infarction Patients
Source: JACC Adv. 2025 Feb 20;4(3):101629. doi: 10.1016/j.jacadv.2025.101629 (PMC11891680; doi:10.1016/j.jacadv.2025.101629)
Supplement: Supplemental Materials [file mmc1.docx]

# Supplemental Figure 1: Odds Ratios of Echocardiogram Evaluation Among T2MI Patients


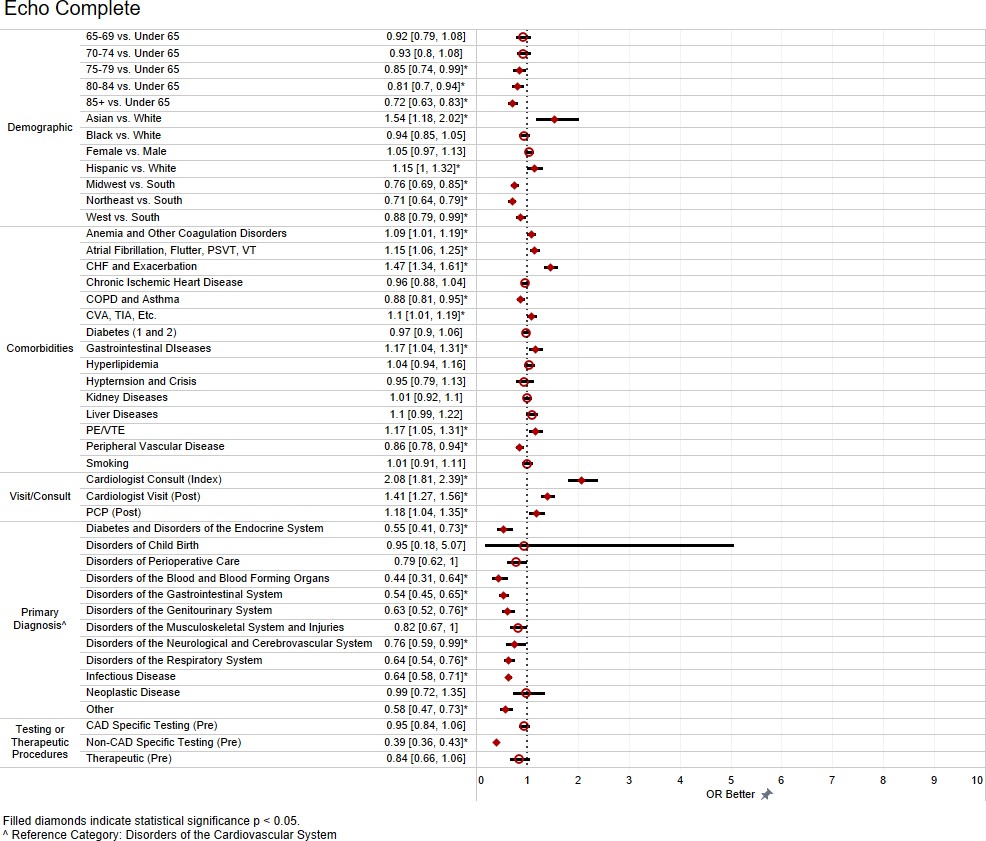


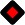
 red diamond = statistically significant.
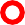
 red circle = statistically insignificant X red cross = null variable Abbreviations: CAD = coronary artery disease. CHF = congestive heart failure. CVA = cerebrovascular accident. COPD = chronic obstructive pulmonary disease. ECHO = echocardiogram. PE = pulmonary embolism. PSVT = paroxysmal supraventricular tachycardia. TIA = transient ischemic attack. VT = ventricular tachycardia. VTE = venothromboembolism.

Cardiac procedures were grouped into three categories: CAD specific (echocardiogram stress test, exercise stress test, nuclear stress test, angiogram, and computed tomography coronary angiogram), non-CAD specific (echocardiogram) and therapeutic (PCI and CABG).

# Supplemental Figure 2: Odds Ratios of An Invasive Coronary Angiogram Among T2MI Patients


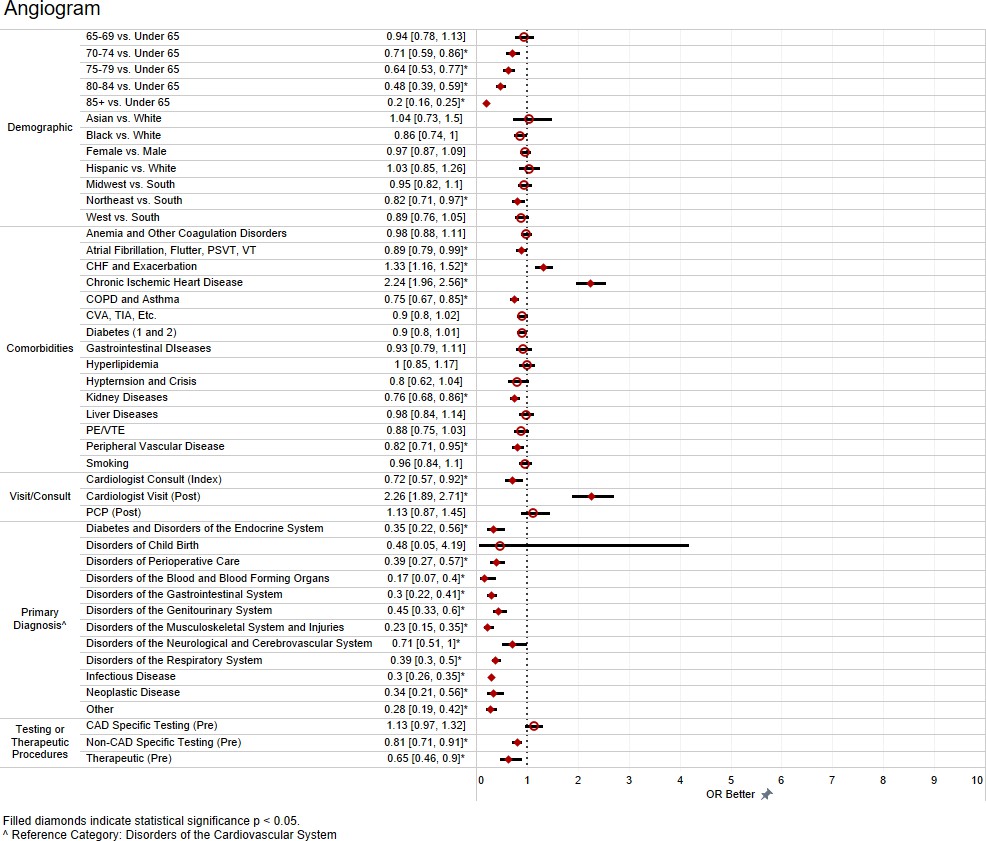


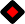
 red diamond = statistically significant.
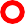
 red circle = statistically insignificant X red cross = null variable Abbreviations: CAD = coronary artery disease. CHF = congestive heart failure. CVA = cerebrovascular accident. COPD = chronic obstructive pulmonary disease. ECHO = echocardiogram. PE = pulmonary embolism. PSVT = paroxysmal supraventricular tachycardia. TIA = transient ischemic attack. VT = ventricular tachycardia. VTE = venothromboembolism.

Cardiac procedures were grouped into three categories: CAD specific (echocardiogram stress test, exercise stress test, nuclear stress test, angiogram, and computed tomography coronary angiogram), non-CAD specific (echocardiogram) and therapeutic (PCI and CABG).

# Supplemental Figure 3: Odds Ratios of Computed Tomography Coronary Angiogram Among T2MI Patients


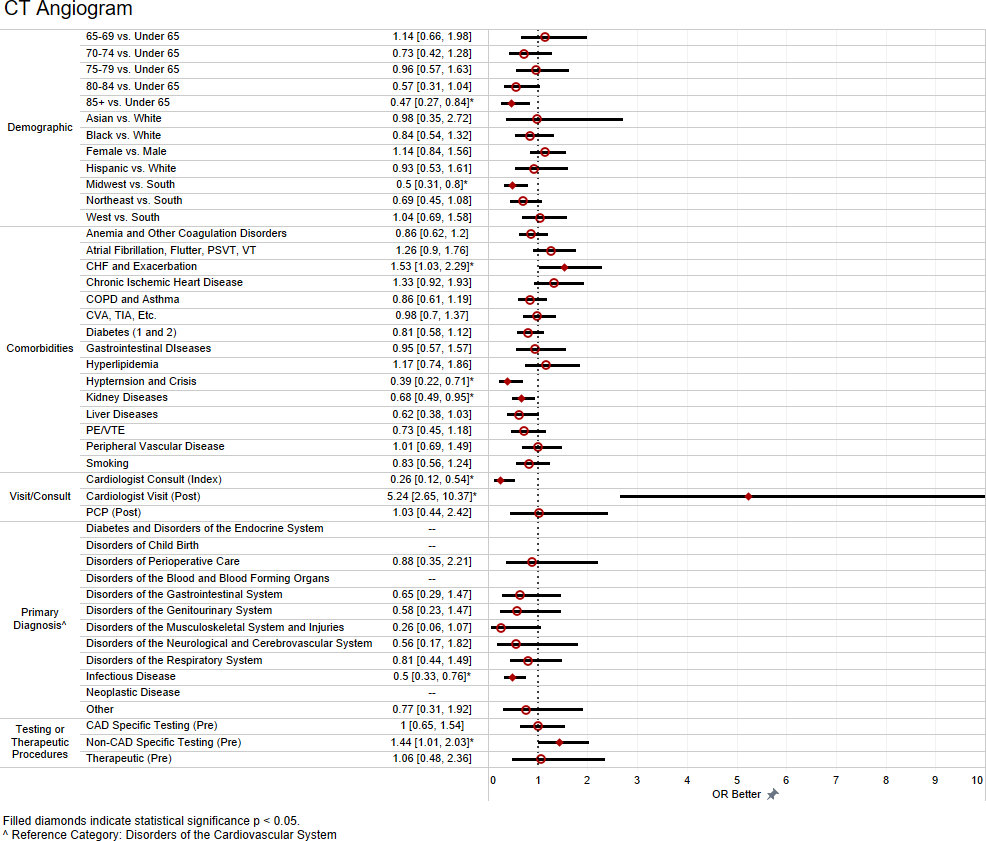


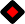
 red diamond = statistically significant.
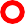
 red circle = statistically insignificant X red cross = null variable Abbreviations: CAD = coronary artery disease. CHF = congestive heart failure. CVA = cerebrovascular accident. COPD = chronic obstructive pulmonary disease. ECHO = echocardiogram. PE = pulmonary embolism. PSVT = paroxysmal supraventricular tachycardia. TIA = transient ischemic attack. VT = ventricular tachycardia. VTE = venothromboembolism.

Cardiac procedures were grouped into three categories: CAD specific (echocardiogram stress test, exercise stress test, nuclear stress test, angiogram, and computed tomography coronary angiogram), non-CAD specific (echocardiogram) and therapeutic (PCI and CABG).

# Supplemental Figure 4: Odds Ratios of An Echocardiogram Stress Test Among T2MI Patients


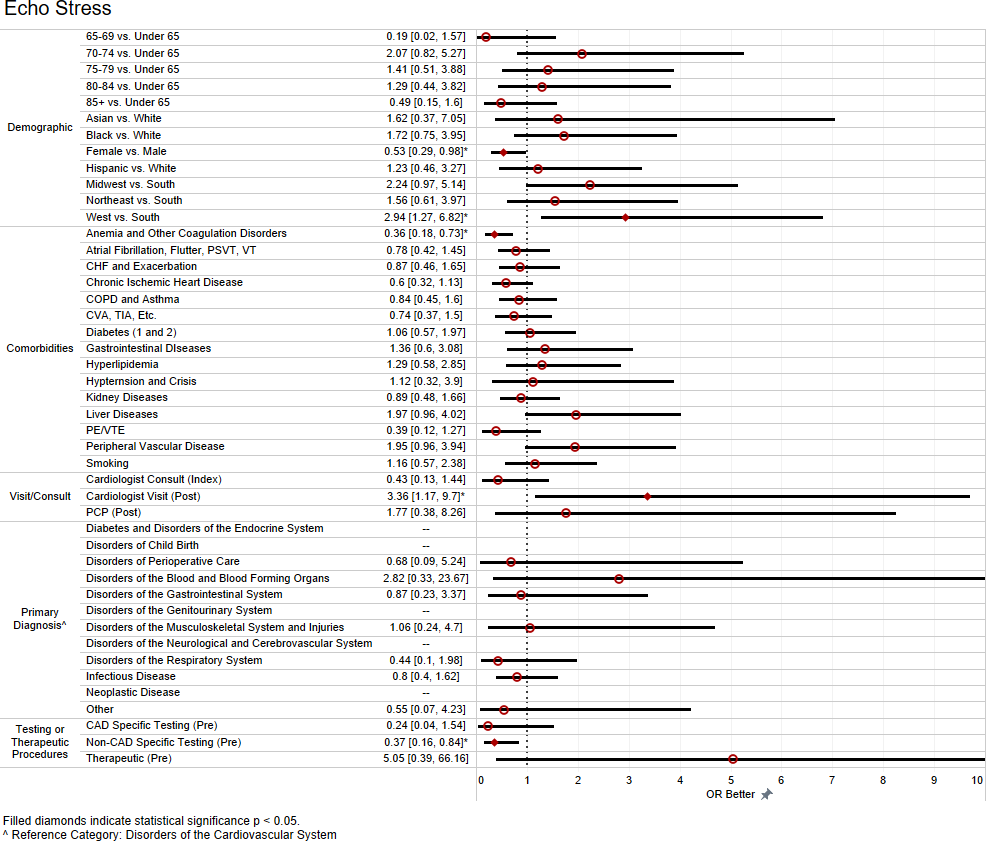


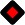
 red diamond = statistically significant.
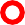
 red circle = statistically insignificant X red cross = null variable Abbreviations: CAD = coronary artery disease. CHF = congestive heart failure. CVA = cerebrovascular accident. COPD = chronic obstructive pulmonary disease. ECHO = echocardiogram. PE = pulmonary embolism. PSVT = paroxysmal supraventricular tachycardia. TIA = transient ischemic attack. VT = ventricular tachycardia. VTE = venothromboembolism.

Cardiac procedures were grouped into three categories: CAD specific (echocardiogram stress test, exercise stress test, nuclear stress test, angiogram, and computed tomography coronary angiogram), non-CAD specific (echocardiogram) and therapeutic (PCI and CABG).

# Supplemental Figure 5: Odds Ratios of Being Evaluated By An Exercise Stress Test Among T2MI Patients


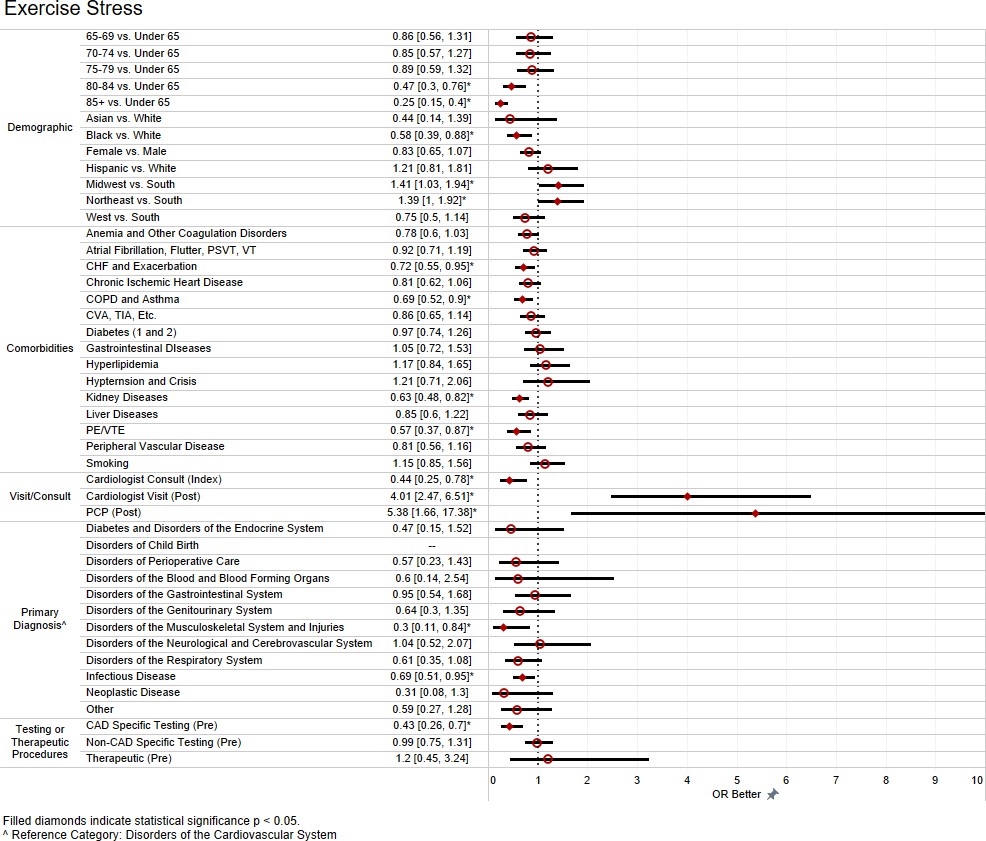


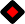
 red diamond = statistically significant.
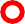
 red circle = statistically insignificant X red cross = null variable Abbreviations: CAD = coronary artery disease. CHF = congestive heart failure. CVA = cerebrovascular accident. COPD = chronic obstructive pulmonary disease. ECHO = echocardiogram. PE = pulmonary embolism. PSVT = paroxysmal supraventricular tachycardia. TIA = transient ischemic attack. VT = ventricular tachycardia. VTE = venothromboembolism.

Cardiac procedures were grouped into three categories: CAD specific (echocardiogram stress test, exercise stress test, nuclear stress test, angiogram, and computed tomography coronary angiogram), non-CAD specific (echocardiogram) and therapeutic (PCI and CABG).

# Supplemental Figure 6: Odds Ratios of A Nuclear Stress Test Among T2MI Patients


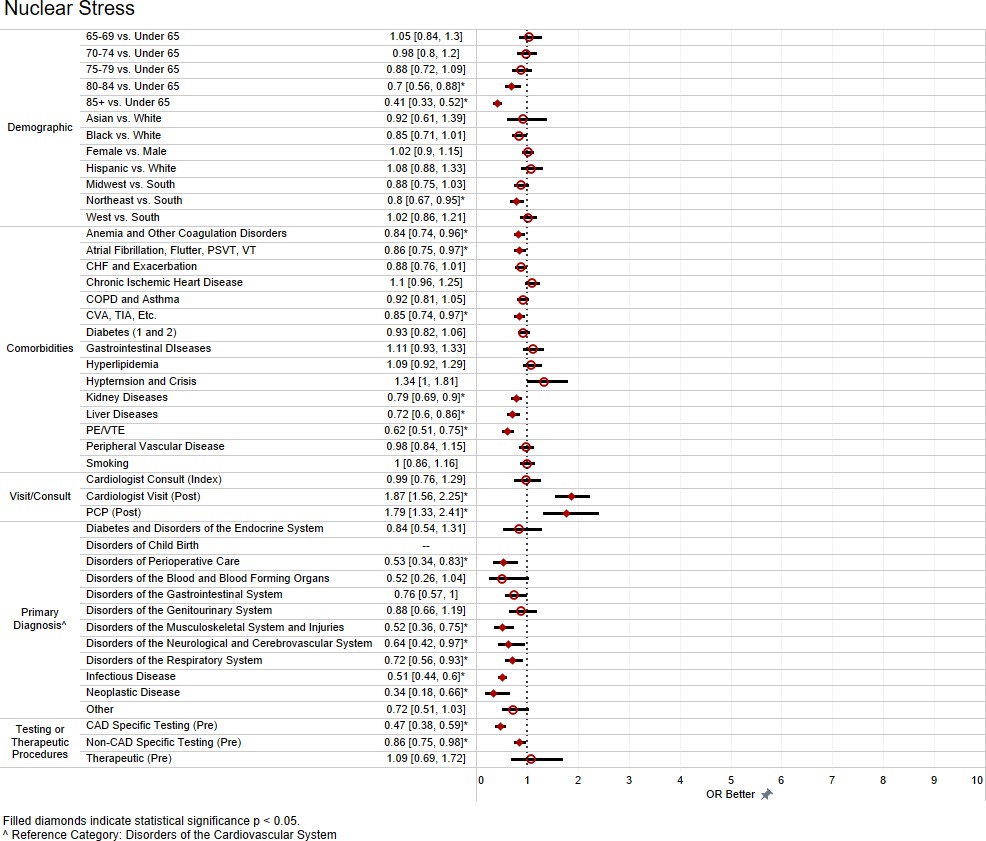


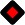
 red diamond = statistically significant.
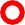
 red circle = statistically insignificant X red cross = null variable Abbreviations: CAD = coronary artery disease. CHF = congestive heart failure. CVA = cerebrovascular accident. COPD = chronic obstructive pulmonary disease. ECHO = echocardiogram. PE = pulmonary embolism. PSVT = paroxysmal supraventricular tachycardia. TIA = transient ischemic attack. VT = ventricular tachycardia. VTE = venothromboembolism.

Cardiac procedures were grouped into three categories: CAD specific (echocardiogram stress test, exercise stress test, nuclear stress test, angiogram, and computed tomography coronary angiogram), non-CAD specific (echocardiogram) and therapeutic (PCI and CABG).

# Supplemental Figure 7: Odds Ratios of Percutaneous Coronary Intervention Among T2MI Patients


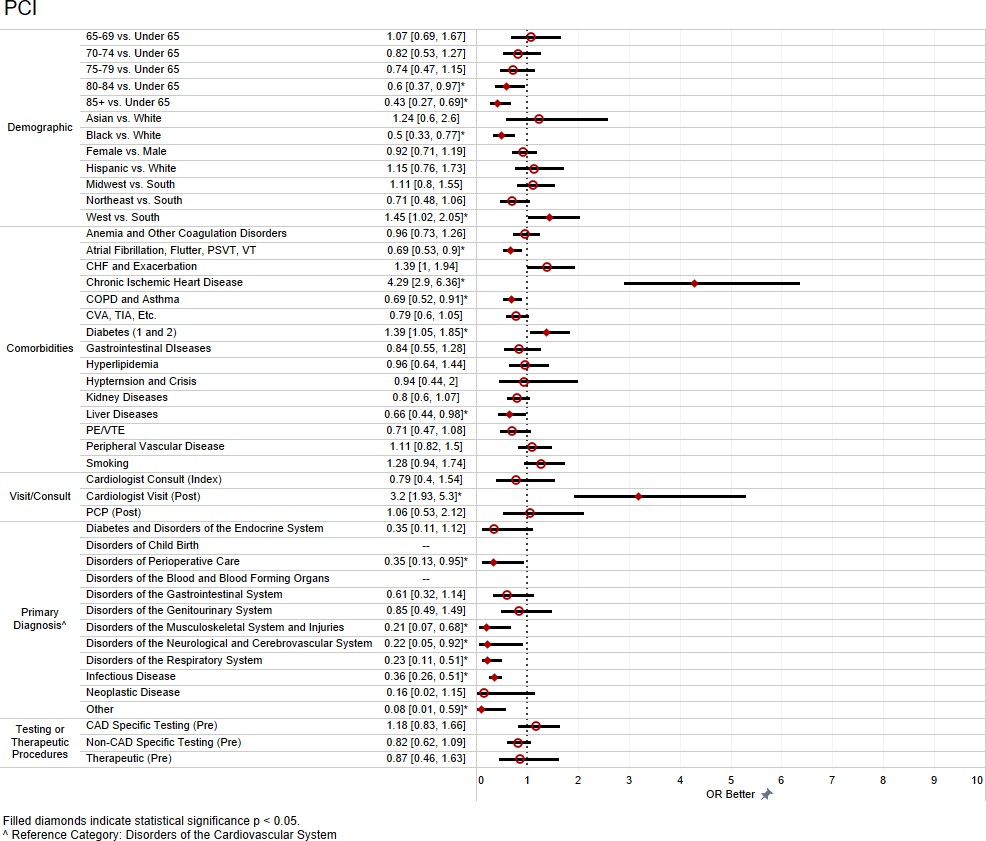


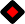
 red diamond = statistically significant.
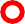
 red circle = statistically insignificant X red cross = null variable Abbreviations: CAD = coronary artery disease. CHF = congestive heart failure. CVA = cerebrovascular accident. COPD = chronic obstructive pulmonary disease. ECHO = echocardiogram. PE = pulmonary embolism. PSVT = paroxysmal supraventricular tachycardia. TIA = transient ischemic attack. VT = ventricular tachycardia. VTE = venothromboembolism.

Cardiac procedures were grouped into three categories: CAD specific (echocardiogram stress test, exercise stress test, nuclear stress test, angiogram, and computed tomography coronary angiogram), non-CAD specific (echocardiogram) and therapeutic (PCI and CABG).

# Supplemental Figure 8: Odds Ratios of Coronary Artery Bypass Graft Among T2MI


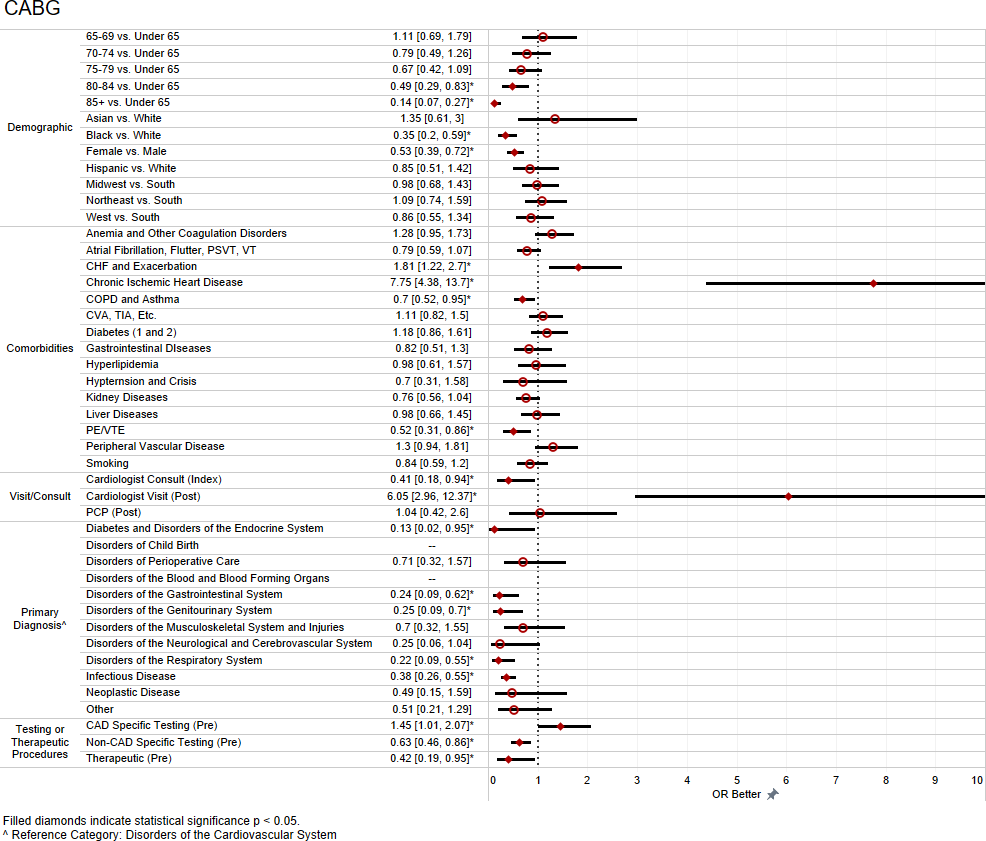


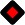
 red diamond = statistically significant.
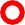
 red circle = statistically insignificant X red cross = null variable Abbreviations: CAD = coronary artery disease. CHF = congestive heart failure. CVA = cerebrovascular accident. COPD = chronic obstructive pulmonary disease. ECHO = echocardiogram. PE = pulmonary embolism. PSVT = paroxysmal supraventricular tachycardia. TIA = transient ischemic attack. VT = ventricular tachycardia. VTE = venothromboembolism.

Cardiac procedures were grouped into three categories: CAD specific (echocardiogram stress test, exercise stress test, nuclear stress test, angiogram, and computed tomography coronary angiogram), non-CAD specific (echocardiogram) and therapeutic (PCI and CABG).

# Supplemental Figure 9: Odds Ratios of A Cardiovascular Physician Consultation Among T2MI


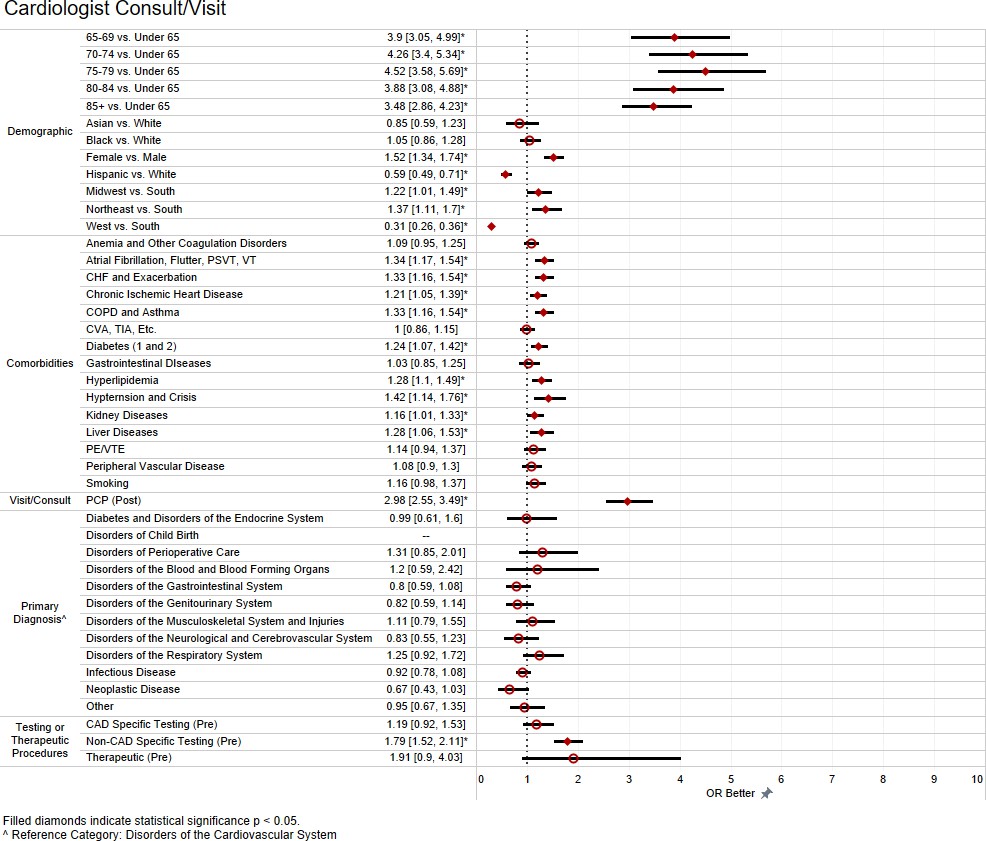


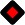
 red diamond = statistically significant.
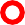
 red circle = statistically insignificant X red cross = null variable Abbreviations: CAD = coronary artery disease. CHF = congestive heart failure. CVA = cerebrovascular accident. COPD = chronic obstructive pulmonary disease. ECHO = echocardiogram. PE = pulmonary embolism. PSVT = paroxysmal supraventricular tachycardia. TIA = transient ischemic attack. VT = ventricular tachycardia. VTE = venothromboembolism.

Cardiac procedures were grouped into three categories: CAD specific (echocardiogram stress test, exercise stress test, nuclear stress test, angiogram, and computed tomography coronary angiogram), non-CAD specific (echocardiogram) and therapeutic (PCI and CABG).

# Supplemental Figure 10: Odds Ratios of ACEi/ARB Prescription Among T2MI Patients


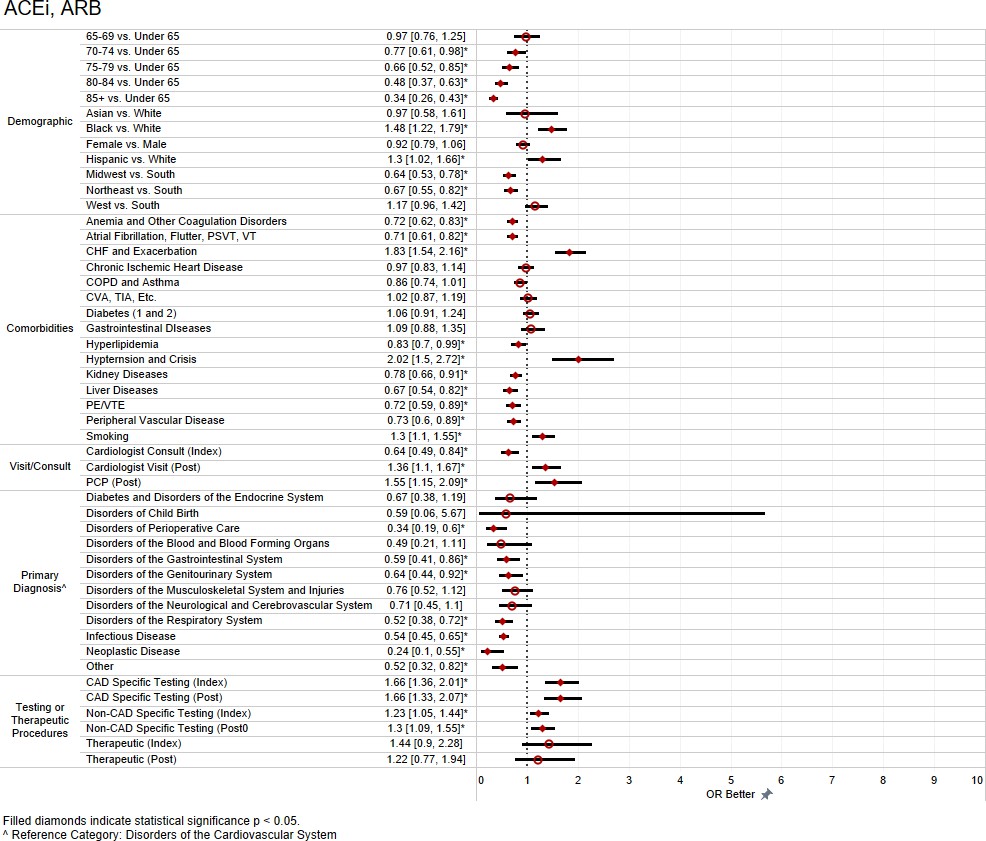


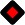
 red diamond = statistically significant.
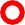
 red circle = statistically insignificant X red cross = null variable Abbreviations: ACEi = angiotensin converting enzyme inhibitor. ARB= angiotensin receptor blocker. CABG = coronary artery bypass graft. CAD = coronary artery disease. CHF = congestive heart failure. CVA = cerebrovascular accident. COPD = chronic obstructive pulmonary disease. ECHO = echocardiogram. PCI = percutaneous coronary intervention. PCP = primary care physician. PE = pulmonary embolism. PSVT = paroxysmal supraventricular tachycardia. TIA = transient ischemic attack. VT = ventricular tachycardia. VTE = venothromboembolism.

Cardiac procedures were grouped into three categories: CAD specific (echocardiogram stress test, exercise stress test, nuclear stress test, angiogram, and computed tomography coronary angiogram), non-CAD specific (echocardiogram) and therapeutic (PCI and CABG).

# Supplemental Figure 11: Odds Ratios Of Aspirin Prescription Among T2MI Patients


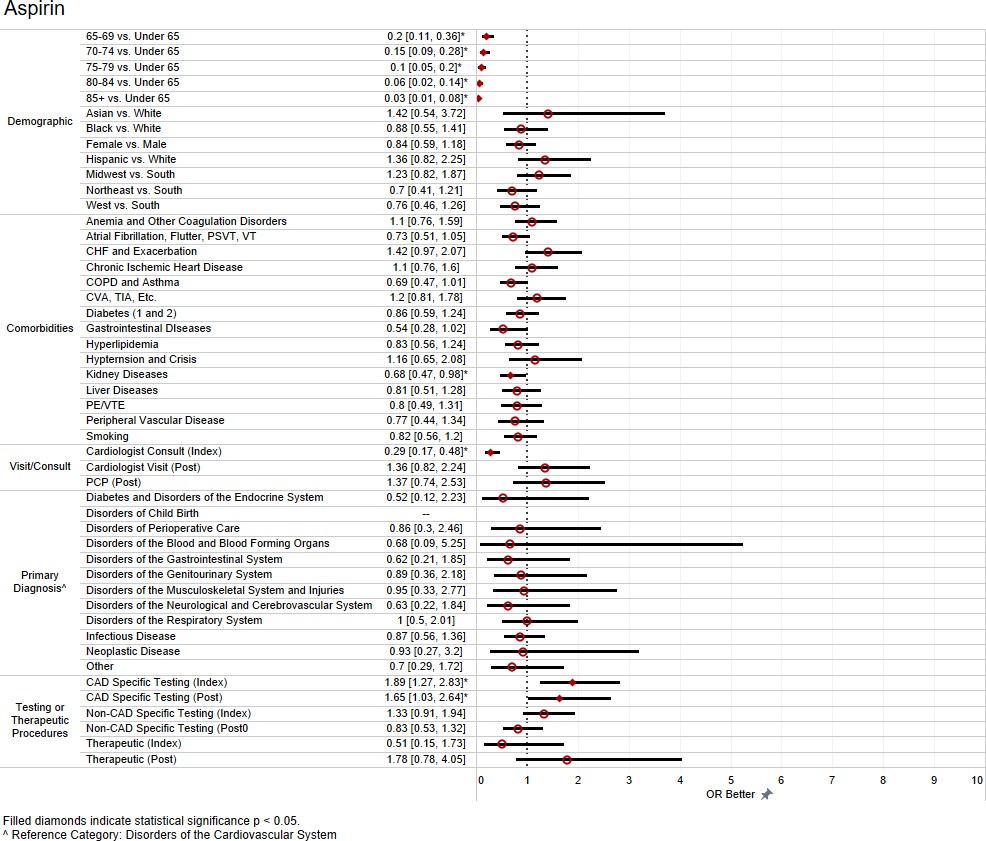


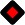
 red diamond = statistically significant.
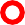
 red circle = statistically insignificant X red cross = null variable Abbreviations: CABG = coronary artery bypass graft. CAD = coronary artery disease. CHF = congestive heart failure. CVA = cerebrovascular accident. COPD = chronic obstructive pulmonary disease. ECHO = echocardiogram. PCI = percutaneous coronary intervention. PCP = primary care physician. PE = pulmonary embolism. PSVT = paroxysmal supraventricular tachycardia. TIA = transient ischemic attack. VT = ventricular tachycardia. VTE = venothromboembolism.

Cardiac procedures were grouped into three categories: CAD specific (echocardiogram stress test, exercise stress test, nuclear stress test, angiogram, and computed tomography coronary angiogram), non-CAD specific (echocardiogram) and therapeutic (PCI and CABG).

# Supplemental Figure 12: Odds Ratios of Beta-Blocker Prescription Among T2MI Patients


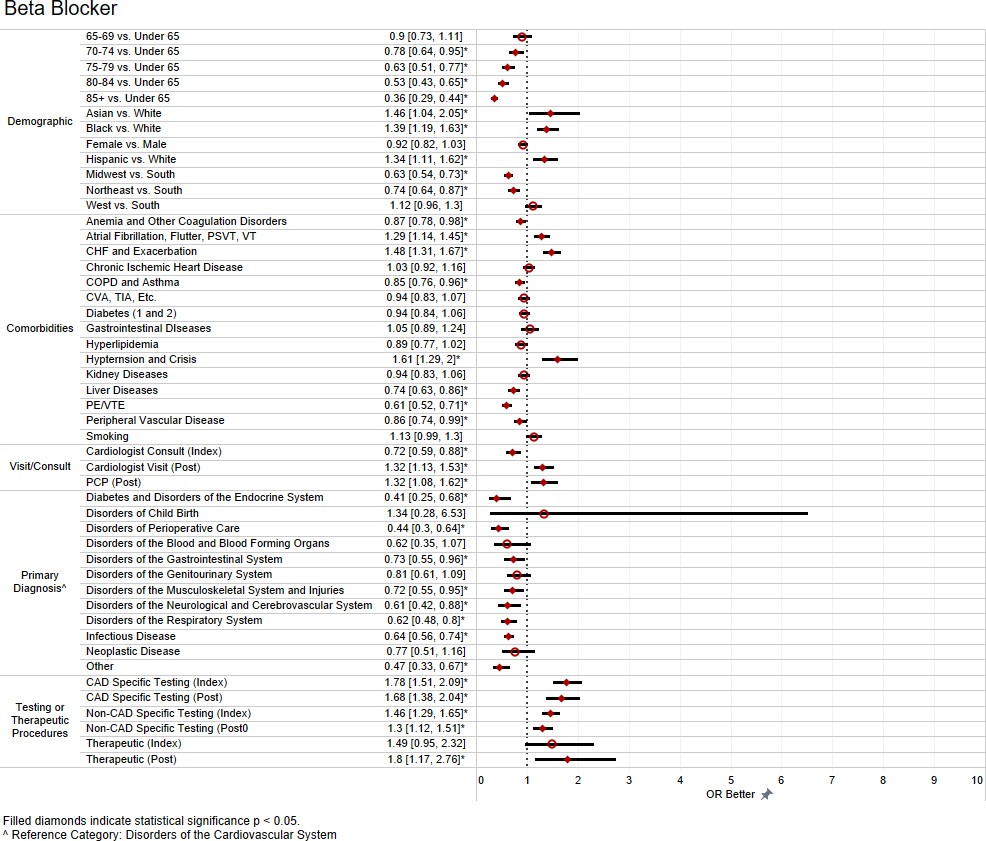


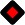
 red diamond = statistically significant.
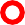
 red circle = statistically insignificant X red cross = null variable Abbreviations: CABG = coronary artery bypass graft. CAD = coronary artery disease. CHF = congestive heart failure. CVA = cerebrovascular accident. COPD = chronic obstructive pulmonary disease. ECHO = echocardiogram. PCI = percutaneous coronary intervention. PCP = primary care physician. PE = pulmonary embolism. PSVT = paroxysmal supraventricular tachycardia. TIA = transient ischemic attack. VT = ventricular tachycardia. VTE = venothromboembolism.

Cardiac procedures were grouped into three categories: CAD specific (echocardiogram stress test, exercise stress test, nuclear stress test, angiogram, and computed tomography coronary angiogram), non-CAD specific (echocardiogram) and therapeutic (PCI and CABG).

# Supplemental Figure 13: Odds Ratios of Sacubitril/Valsartan Prescription Among T2MI Patients


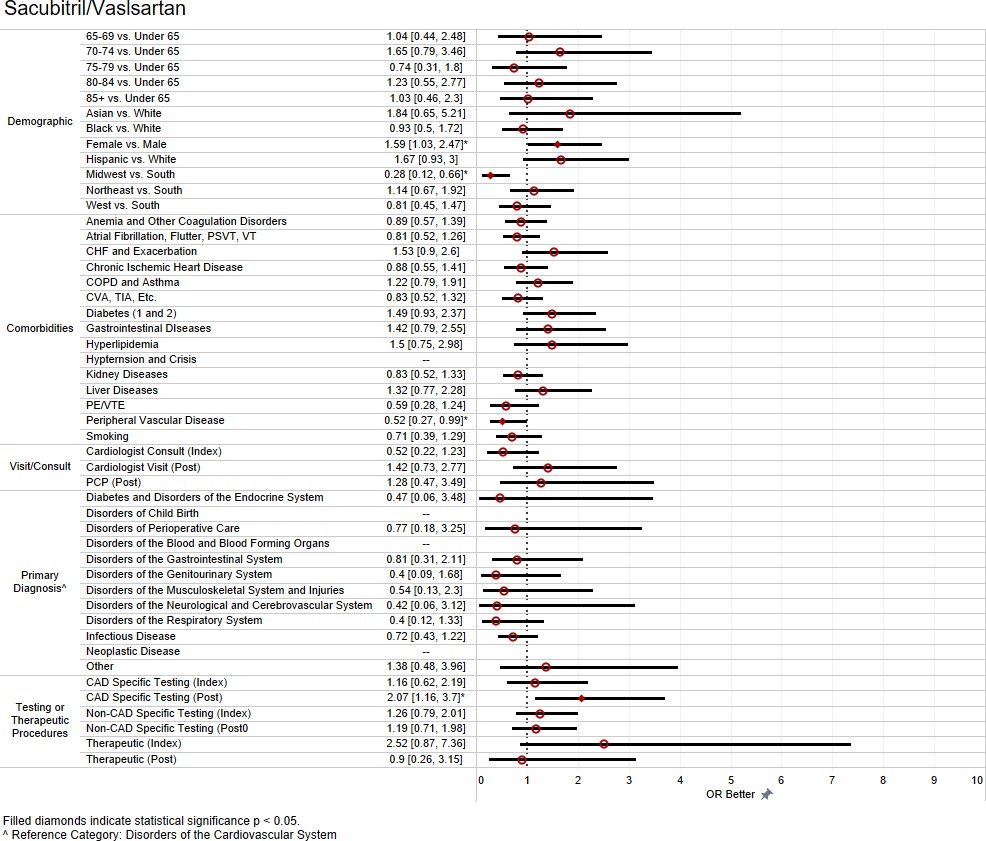


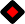
 red diamond = statistically significant.
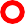
 red circle = statistically insignificant X red cross = null variable Abbreviations: CABG = coronary artery bypass graft. CAD = coronary artery disease. CHF = congestive heart failure. CVA = cerebrovascular accident. COPD = chronic obstructive pulmonary disease. ECHO = echocardiogram. PCI = percutaneous coronary intervention. PCP = primary care physician. PE = pulmonary embolism. PSVT = paroxysmal supraventricular tachycardia. TIA = transient ischemic attack. VT = ventricular tachycardia. VTE = venothromboembolism.

Cardiac procedures were grouped into three categories: CAD specific (echocardiogram stress test, exercise stress test, nuclear stress test, angiogram, and computed tomography coronary angiogram), non-CAD specific (echocardiogram) and therapeutic (PCI and CABG).

# Supplemental Figure 14: Odds Ratios of GLP1-Agoinst/SGLT-i Prescription Among T2MI Patients


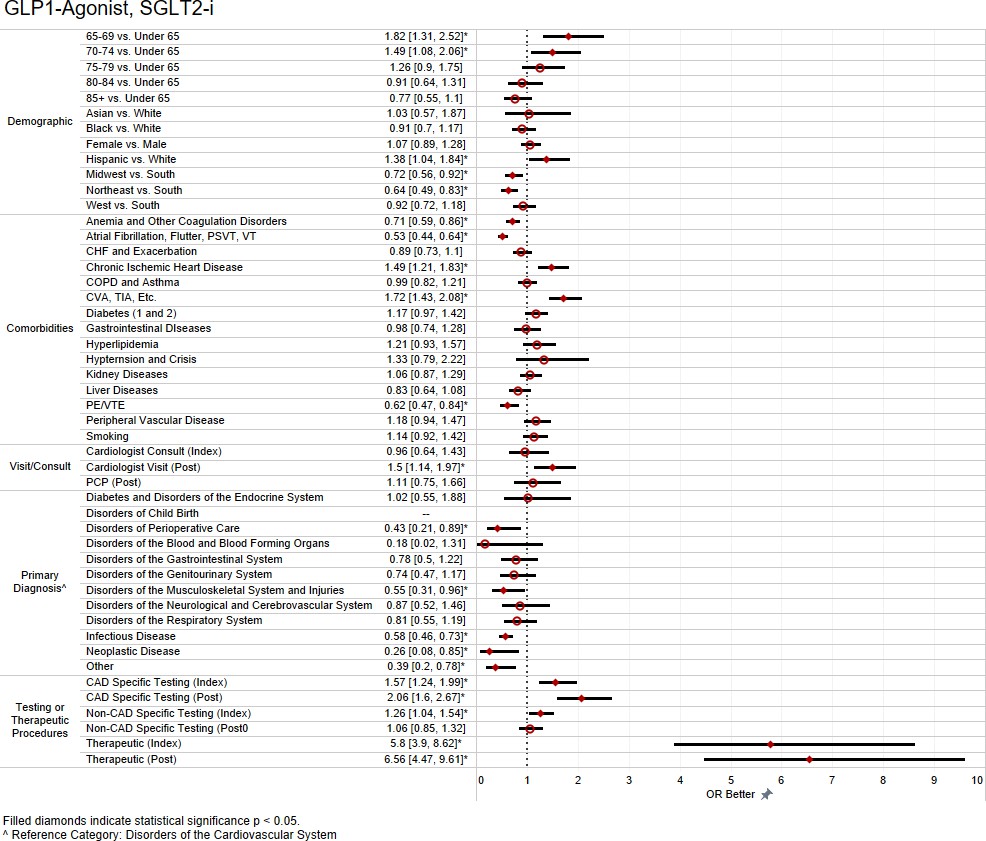


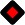
 red diamond = statistically significant.
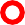
 red circle = statistically insignificant X red cross = null variable Abbreviations: CABG = coronary artery bypass graft. CAD = coronary artery disease. CHF = congestive heart failure. CVA = cerebrovascular accident. COPD = chronic obstructive pulmonary disease. ECHO = echocardiogram. PCI = percutaneous coronary intervention. GLP-1 = glucagon like peptide-1. PCP = primary care physician. PE = pulmonary embolism. PSVT = paroxysmal supraventricular tachycardia. SGLT2-i = sodium glucose co-transporter 2 inhibitor. TIA = transient ischemic attack. VT = ventricular tachycardia. VTE = venothromboembolism.

Cardiac procedures were grouped into three categories: CAD specific (echocardiogram stress test, exercise stress test, nuclear stress test, angiogram, and computed tomography coronary angiogram), non-CAD specific (echocardiogram) and therapeutic (PCI and CABG).

# Supplemental Figure 15: Odds Ratios of P2Y12 Inhibitor Prescription Among T2MI Patients


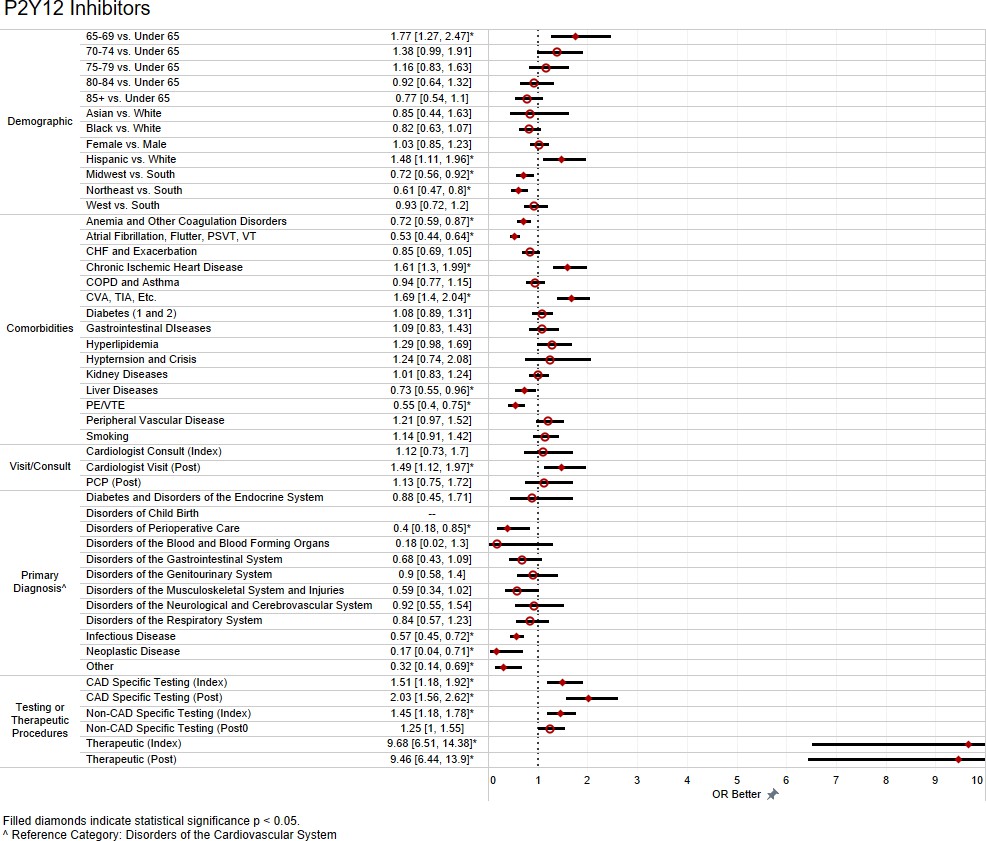


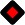
 red diamond = statistically significant.
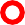
 red circle = statistically insignificant X red cross = null variable Abbreviations: CABG = coronary artery bypass graft. CAD = coronary artery disease. CHF = congestive heart failure. CVA = cerebrovascular accident. COPD = chronic obstructive pulmonary disease. ECHO = echocardiogram. PCI = percutaneous coronary intervention. P2Y12 = purigenic receptor inhibitor. PCP = primary care physician. PE = pulmonary embolism. PSVT = paroxysmal supraventricular tachycardia. TIA = transient ischemic attack. VT = ventricular tachycardia. VTE = venothromboembolism.

Cardiac procedures were grouped into three categories: CAD specific (echocardiogram stress test, exercise stress test, nuclear stress test, angiogram, and computed tomography coronary angiogram), non-CAD specific (echocardiogram) and therapeutic (PCI and CABG).

# Supplemental Figure 16: Odds Ratios of PCSK9/Ezetimibe Prescription Among T2MI Patients


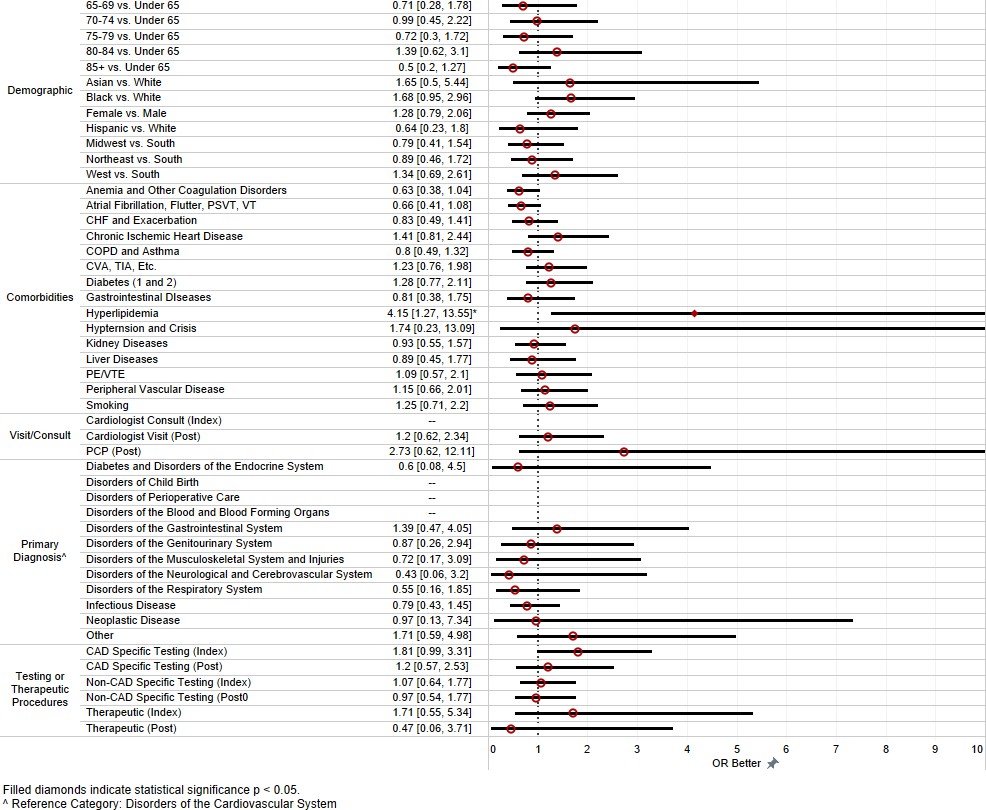


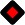
 red diamond = statistically significant.
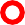
 red circle = statistically insignificant X red cross = null variable Abbreviations: CABG = coronary artery bypass graft. CAD = coronary artery disease. CHF = congestive heart failure. CVA = cerebrovascular accident. COPD = chronic obstructive pulmonary disease. ECHO = echocardiogram. PCI = percutaneous coronary intervention. PCP = primary care physician. PCSK9 = paraprotein convertase subtilisin/kexin type 9. PE = pulmonary embolism. PSVT = paroxysmal supraventricular tachycardia. TIA = transient ischemic attack. VT = ventricular tachycardia. VTE = venothromboembolism.

Cardiac procedures were grouped into three categories: CAD specific (echocardiogram stress test, exercise stress test, nuclear stress test, angiogram, and computed tomography coronary angiogram), non-CAD specific (echocardiogram) and therapeutic (PCI and CABG).

# Supplemental Figure 17: Odds Ratios of Statin Prescription Among T2MI Patients


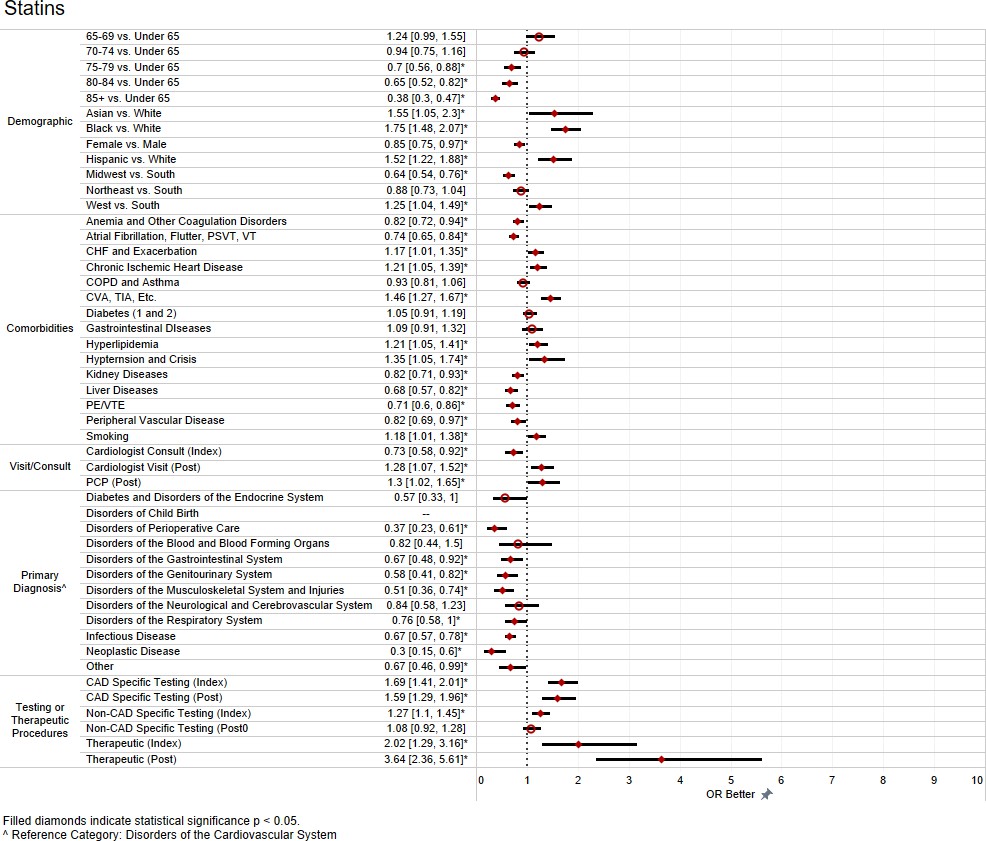


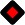
 red diamond = statistically significant.
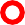
 red circle = statistically insignificant X red cross = null variable Abbreviations: CABG = coronary artery bypass graft. CAD = coronary artery disease. CHF = congestive heart failure. CVA = cerebrovascular accident. COPD = chronic obstructive pulmonary disease. ECHO = echocardiogram. PCI = percutaneous coronary intervention. PCP = primary care physician. PE = pulmonary embolism. PSVT = paroxysmal supraventricular tachycardia. TIA = transient ischemic attack. VT = ventricular tachycardia. VTE = venothromboembolism.

Cardiac procedures were grouped into three categories: CAD specific (echocardiogram stress test, exercise stress test, nuclear stress test, angiogram, and computed tomography coronary angiogram), non-CAD specific (echocardiogram) and therapeutic (PCI and CABG).

# Supplemental Table 1: Individual ICD Diagnosis Associated With Each Primary Diagnostic Group

| Blood and Blood Forming  Organs | | | Cardiovascular | | | Gastrointestinal | | | Endocrine | | |
| --- | --- | --- | --- | --- | --- | --- | --- | --- | --- | --- | --- |
|  | Nutritional anemia |  |  | Chronic rheumatic heart  disease |  |  | Diseases of mouth;  excluding dental |  |  | Thyroid disorders |  |
|  | *Hemolytic* anemia |  |  |  |  |  |  |  |  | Diabetes mellitus  without complication |  |
|  |  |  |  | Acute rheumatic heart  disease |  |  | Esophageal disorders |  |  |  |  |
|  | Aplastic anemia |  |  |  |  |  |  |  |  |  |  |
|  |  |  |  |  |  |  | Gastroduodenal ulcer |  |  | Diabetes mellitus with  complication |  |
|  | Acute posthemorrhagic  anemia |  |  |  |  |  |  |  |  |  |  |
|  |  |  |  | Nonrheumatic and unspecified valve  disorders |  |  |  |  |  |  |  |
|  |  |  |  |  |  |  | Gastrointestinal and  biliary perforation |  |  |  |  |
|  |  |  |  |  |  |  |  |  |  | Nutritional deficiencies |  |
|  | Sickle cell trait/anemia |  |  |  |  |  |  |  |  |  |  |
|  |  |  |  |  |  |  | Gastritis and duodenitis |  |  | Malnutrition |  |
|  | Coagulation and  hemorrhagic disorders |  |  | Endocarditis and endocardial disease |  |  |  |  |  |  |  |
|  |  |  |  |  |  |  | Other specified and unspecified disorders of stomach and  duodenum |  |  | Obesity |  |
|  | Diseases of white blood  cells |  |  | Myocarditis and cardiomyopathy |  |  |  |  |  | Disorders of lipid  metabolism |  |
|  | Immunity disorders |  |  | Pericarditis and pericardial disease |  |  |  |  |  | Pituitary disorders |  |
|  |  |  |  |  |  |  | Appendicitis and other  appendiceal conditions |  |  |  |  |
|  | Other specified and unspecified  hematologic conditions |  |  |  |  |  |  |  |  | Other specified and unspecified endocrine  disorders |  |
|  |  |  |  | Essential hypertension |  |  |  |  |  |  |  |
|  |  |  |  |  |  |  | Abdominal hernia |  |  |  |  |
|  |  |  |  | Hypertension with complications and secondary  hypertension |  |  |  |  |  |  |  |
|  | | |  |  |  |  | Regional enteritis and  ulcerative colitis |  |  | Other specified and unspecified nutritional and metabolic  disorders |  |
|  |  |  |  |  |  |  | Intestinal obstruction and ileus |  |  |  |  |
|  |  |  |  | Acute myocardial  infarction |  |  |  |  |  |  |  |
|  |  |  |  |  |  |  | Diverticulosis and  diverticulitis |  |  | | |
|  |  |  |  | Complications of acute  myocardial infarction |  |  |  |  |  |  |  |
|  |  |  |  |  |  |  | Hemorrhoids |  |  |  |  |
|  |  |  |  | Coronary atherosclerosis and other heart disease |  |  | Anal and rectal  conditions |  |  |  |  |
|  |  |  |  |  |  |  | Biliary tract disease |  |  |  |  |
|  |  |  |  | Nonspecific chest pain |  |  | Hepatic failure |  |  |  |  |
|  |  |  |  | Acute pulmonary  embolism |  |  | Other specified and  unspecified liver disease |  |  |  |  |
|  |  |  |  | Pulmonary heart  disease |  |  |  |  |  |  |  |
|  |  |  |  |  |  |  | Pancreatic disorders  (excluding diabetes) |  |  |  |  |
|  |  |  |  | Other and ill-defined  heart disease |  |  |  |  |  |  |  |
|  |  |  |  |  |  |  | Gastrointestinal  hemorrhage |  |  |  |  |
|  |  |  |  | Conduction disorders |  |  |  |  |  |  |  |
|  |  |  |  | Cardiac dysrhythmias |  |  | Noninfectious  gastroenteritis |  |  |  |  |
|  |  |  |  | Cardiac arrest and  ventricular fibrillation |  |  |  |  |  |  |  |
|  |  |  |  |  |  |  | Noninfectious hepatitis |  |  |  |  |
|  |  |  |  | Heart failure |  |  | Other specified and unspecified  gastrointestinal disorders |  |  |  |  |
|  |  |  |  | Cerebral infarction |  |  |  |  |  |  |  |
|  |  |  |  | Peripheral and visceral  vascular disease |  |  |  |  |  |  |  |
|  |  |  |  |  |  |  | Digestive congenital  anomalies |  |  |  |  |
|  |  |  |  | Arterial dissections |  |  |  |  |  |  |  |
|  |  |  |  | Hypotension |  |  | Nausea and vomiting |  |  |  |  |
|  |  |  |  | Other specified and  unspecified circulatory disease |  |  | Dysphagia |  |  |  |  |
|  |  |  |  |  |  |  | Abdominal pain and other digestive/abdomen signs and  symptoms |  |  |  |  |
|  |  |  |  | Acute phlebitis; thrombophlebitis and  thromboembolism |  |  |  |  |  |  |  |
|  |  |  |  |  |  |  |  |  |  |  |  |

|  |  | Varicose veins of lower  extremity |  |  | Malaise and fatigue |  |  |
| --- | --- | --- | --- | --- | --- | --- | --- |
|  |  |  |  |  | | |  |
|  |  | Postthrombotic syndrome and venous insufficiency/hypertensi  on |  |  |  |  |  |
|  |  | Other specified diseases  of veins and lymphatics |  |  |  |  |  |
|  |  | Cardiac and circulatory congenital anomalies |  |  |  |  |  |
|  |  | Circulatory signs and  symptoms |  |  |  |  |  |

Clinical diagnoses were aggregated using the Clinical Classifications Software Refined (CCSR). As a component of the Healthcare Cost and Utilization Project (HCUP), the CCSR groups over 700,000 ICD-10-CM codes into over 530 clinical diagnostic categories.

# Supplemental Table 2: Individual ICD Diagnosis Associated With Each Primary Diagnostic Group

| Genitourinary | | | Infections | | | Musculoskeletal and  Injury | | | Neoplams | | |
| --- | --- | --- | --- | --- | --- | --- | --- | --- | --- | --- | --- |
|  | Nephritis; nephrosis;  renal sclerosis |  |  | Intestinal infection |  |  | Fracture of head and  neck, initial encounter |  |  | Encounter for  antineoplastic therapies |  |
|  |  |  |  | Peritonitis and intra-  abdominal abscess |  |  |  |  |  |  |  |
|  | Acute and unspecified  renal failure |  |  |  |  |  | Fracture of the spine and back, initial encounter |  |  | Neoplasm-related  encounters |  |
|  |  |  |  | Exposure, encounters, screening or contact with  infectious disease |  |  |  |  |  |  |  |
|  | Chronic kidney disease |  |  |  |  |  |  |  |  | Head and neck cancers  - lip and oral cavity |  |
|  | Calculus of urinary tract |  |  |  |  |  | Fracture of torso, initial  encounter |  |  |  |  |
|  |  |  |  |  |  |  |  |  |  | Head and neck cancers  - throat |  |
|  | Other specified and unspecified diseases of kidney and  ureters |  |  |  |  |  |  |  |  |  |  |
|  |  |  |  | Urinary tract infections |  |  | Fracture of the upper  limb, initial encounter |  |  |  |  |
|  |  |  |  |  |  |  |  |  |  | Head and neck cancers  - salivary gland |  |
|  |  |  |  | Tuberculosis |  |  |  |  |  |  |  |
|  |  |  |  |  |  |  | Fracture of the lower  limb (except hip), initial encounter |  |  |  |  |
|  |  |  |  | Septicemia |  |  |  |  |  | Head and neck cancers  - nasopharyngeal |  |
|  | Hematuria |  |  |  |  |  |  |  |  |  |  |
|  |  |  |  | Bacterial infections |  |  |  |  |  |  |  |
|  | Hyperplasia of prostate |  |  | Fungal infections |  |  | Fracture of the neck of the femur (hip), initial  encounter |  |  | Head and neck cancers  - hypopharyngeal |  |
|  | Inflammatory conditions of male  genital organs |  |  |  |  |  |  |  |  |  |  |
|  |  |  |  | HIV infection |  |  |  |  |  |  |  |
|  |  |  |  |  |  |  |  |  |  | Head and neck cancers  - laryngeal |  |
|  |  |  |  | Hepatitis |  |  | Dislocations, initial encounter |  |  |  |  |
|  | Erectile dysfunction |  |  | Viral infection |  |  |  |  |  | Head and neck cancers  - tonsils |  |
|  | Other specified male genital disorders |  |  | Parasitic, other specified and  unspecified infections |  |  | Traumatic brain injury  (TBI); concussion, initial encounter |  |  |  |  |
|  |  |  |  |  |  |  |  |  |  | Head and neck cancers  - all other types |  |
|  | Nonmalignant breast conditions |  |  |  |  |  |  |  |  |  |  |
|  |  |  |  | Infective arthritis |  |  | Spinal cord injury  (SCI), initial encounter |  |  | Cardiac cancers |  |
|  | Inflammatory diseases  of female pelvic organs |  |  | Osteomyelitis |  |  |  |  |  | Gastrointestinal cancers  - esophagus |  |
|  |  |  |  |  |  |  | Internal organ injury,  initial encounter |  |  |  |  |
|  |  |  |  | Musculoskeletal  abscess |  |  |  |  |  |  |  |
|  | Prolapse of female  genital organs |  |  |  |  |  |  |  |  | Gastrointestinal cancers  - stomach |  |
|  |  |  |  |  |  |  | Open wounds of head and neck, initial encounter |  |  |  |  |
|  |  |  |  | Meningitis |  |  |  |  |  |  |  |
|  | Menstrual disorders |  |  |  |  |  |  |  |  | Gastrointestinal cancers  - small intestine |  |
|  |  |  |  | Encephalitis |  |  |  |  |  |  |  |
|  | Menopausal disorders |  |  |  |  |  |  |  |  |  |  |
|  |  |  |  | CNS abscess |  |  | Open wounds to limbs,  initial encounter |  |  | Gastrointestinal cancers  - colorectal |  |
|  | Other specified female  genital disorders |  |  |  |  |  |  |  |  |  |  |
|  |  |  |  | Pneumonia (except that  caused by tuberculosis) |  |  |  |  |  |  |  |
|  |  |  |  |  |  |  | Open wounds of trunk,  initial encounter |  |  |  |  |
|  |  |  |  |  |  |  |  |  |  | Gastrointestinal cancers  - anus |  |
|  | Genitourinary signs and  symptoms |  |  |  |  |  |  |  |  |  |  |
|  |  |  |  | Influenza |  |  |  |  |  |  |  |
|  |  |  |  |  |  |  | Injury to blood vessels,  initial encounter |  |  |  |  |
|  |  |  |  |  |  |  |  |  |  | Gastrointestinal cancers  - liver |  |
|  | | |  | Acute bronchitis |  |  |  |  |  |  |  |
|  |  |  |  | Other specified upper  respiratory infections |  |  | Superficial injury; contusion, initial encounter |  |  |  |  |
|  |  |  |  |  |  |  |  |  |  | Gastrointestinal cancers  - bile duct |  |
|  |  |  |  | Aspiration pneumonitis |  |  |  |  |  |  |  |
|  |  |  |  |  |  |  |  |  |  | Gastrointestinal cancers  - gallbladder |  |
|  |  |  |  | Skin and subcutaneous tissue infections |  |  | Burn and corrosion,  initial encounter |  |  |  |  |
|  |  |  |  |  |  |  |  |  |  | Gastrointestinal cancers  - peritoneum |  |
|  |  |  |  | | |  | Toxic effects, initial  encounter |  |  |  |  |
|  |  |  |  |  |  |  |  |  |  | Gastrointestinal cancers  - all other types |  |
|  |  |  |  |  |  |  | Sprains and strains,  initial encounter |  |  |  |  |
|  |  |  |  |  |  |  |  |  |  | Respiratory cancers |  |
|  |  |  |  |  |  |  | Injury to nerves, muscles and tendons, initial encounter |  |  |  |  |
|  |  |  |  |  |  |  |  |  |  | Bone cancer |  |
|  |  |  |  |  |  |  |  |  |  | Sarcoma |  |
|  |  |  |  |  |  |  | Other specified injury |  |  | Skin cancers - melanoma |  |
|  |  |  |  |  |  |  | Other unspecified  injury |  |  |  |  |
|  |  |  |  |  |  |  |  |  |  | Skin cancers - basal cell  carcinoma |  |
|  |  |  |  |  |  |  | Fracture of head and neck, subsequent |  |  |  |  |
|  |  |  |  |  |  |  |  |  |  | Skin cancers - |  |

|  |  |  | encounter |  |  | squamous cell |  |
| --- | --- | --- | --- | --- | --- | --- | --- |
|  |  |  |  |  |  | carcinoma |  |
|  |  |  | Fracture of the spine and back, subsequent  encounter |  |  |  |  |
|  |  |  |  |  |  | Breast cancer - ductal  carcinoma in situ (DCIS) |  |
|  |  |  | Fracture of the upper  limb, subsequent encounter |  |  |  |  |
|  |  |  |  |  |  | Breast cancer - all other types |  |
|  |  |  | Fracture of lower limb (except hip), subsequent encounter |  |  | Female reproductive  system cancers - uterus |  |
|  |  |  |  |  |  | Female reproductive system cancers - cervix |  |
|  |  |  | Fracture of the neck of the femur (hip), subsequent  encounter |  |  |  |  |
|  |  |  |  |  |  | Female reproductive  system cancers - ovary |  |
|  |  |  |  |  |  | Female reproductive system cancers -  fallopian tube |  |
|  |  |  | Traumatic brain injury (TBI); concussion, subsequent  encounter |  |  |  |  |
|  |  |  |  |  |  | Female reproductive  system cancers - endometrium |  |
|  |  |  | Burns and corrosion,  subsequent encounter |  |  |  |  |
|  |  |  |  |  |  | Female reproductive  system cancers - vulva |  |
|  |  |  | Injury, sequela |  |  |  |  |
|  |  |  |  |  |  | Female reproductive  system cancers - vagina |  |
|  |  |  | Musculoskeletal  congenital conditions |  |  |  |  |
|  |  |  |  |  |  | Female reproductive system cancers - all  other types |  |
|  |  |  | Rheumatoid arthritis  and related disease |  |  |  |  |
|  |  |  | Osteoarthritis |  |  |  |  |
|  |  |  |  |  |  | Male reproductive  system cancers - prostate |  |
|  |  |  | Other specified joint disorders |  |  |  |  |
|  |  |  | Tendon and synovial  disorders |  |  | Urinary system cancers  - bladder |  |
|  |  |  | Musculoskeletal pain,  not low back pain |  |  | Urinary system cancers  - ureter and renal pelvis |  |
|  |  |  | Spondylopathies/spond  yloarthropathy (including  infective) |  |  | Urinary system cancers  - kidney |  |
|  |  |  |  |  |  | Urinary system cancers  - urethra |  |
|  |  |  | Osteoporosis |  |  |  |  |
|  |  |  |  |  |  | Nervous system cancers |  |
|  |  |  | Pathological fracture,  initial encounter |  |  | - brain |  |
|  |  |  |  |  |  | Endocrine system |  |
|  |  |  | Stress fracture, initial encounter |  |  | cancers - thyroid |  |
|  |  |  |  |  |  | Endocrine system |  |
|  |  |  | Stress fracture,  subsequent encounter |  |  | cancers - pancreas |  |
|  |  |  |  |  |  | Endocrine system |  |
|  |  |  | Scoliosis and other  postural dorsopathic |  |  | cancers - thymus |  |
|  |  |  |  |  |  | Hodgkin lymphoma |  |
|  |  |  | deformities |  |  |  |  |
|  |  |  |  |  |  | Non-Hodgkin lymphoma |  |
|  |  |  | Acquired deformities  (excluding foot) |  |  |  |  |
|  |  |  |  |  |  | Leukemia - acute lymphoblastic leukemia  (ALL) |  |
|  |  |  | Systemic lupus erythematosus and connective tissue  disorders |  |  |  |  |
|  |  |  |  |  |  | Leukemia - acute myeloid leukemia  (AML) |  |
|  |  |  | Other specified  connective tissue |  |  |  |  |
|  |  |  |  |  |  |  |  |

|  |  |  | disease |  |  | Leukemia - chronic lymphocytic leukemia  (CLL) |  |
| --- | --- | --- | --- | --- | --- | --- | --- |
|  |  |  | Muscle disorders |  |  |  |  |
|  |  |  | Aseptic necrosis and  osteonecrosis |  |  |  |  |
|  |  |  |  |  |  | Leukemia - chronic myeloid leukemia  (CML) |  |
|  |  |  | Neurogenic/neuropathic arthropathy |  |  |  |  |
|  |  |  |  |  |  | Leukemia - all other  types |  |
|  |  |  | Gout |  |  |  |  |
|  |  |  | Low back pain |  |  | Multiple myeloma |  |
|  |  |  | | |  | Malignant neuroendocrine tumors |  |
|  |  |  |  |  |  | Mesothelioma |  |
|  |  |  |  |  |  | Myelodysplastic syndrome (MDS) |  |
|  |  |  |  |  |  | Cancer of other sites |  |
|  |  |  |  |  |  | Secondary malignancies |  |
|  |  |  |  |  |  | Malignant neoplasm,  unspecified |  |
|  |  |  |  |  |  | Neoplasms of unspecified nature or  uncertain behavior |  |
|  |  |  |  |  |  | Benign neoplasms |  |
|  |  |  |  |  |  | Conditions due to neoplasm or the  treatment of neoplasm |  |

Clinical diagnoses were aggregated using the Clinical Classifications Software Refined (CCSR). As a component of the Healthcare Cost and Utilization Project (HCUP), the CCSR groups over 700,000 ICD-10-CM codes into over 530 clinical diagnostic categories.

# Supplemental Table 3: Individual ICD Diagnosis Associated With Each Primary Diagnostic Group

| Neurological and Cerebrovascular | Perioperative Care | Child Birth | Respiratory | Other |
| --- | --- | --- | --- | --- |

|  | Acute hemorrhagic cerebrovascular  disease |  |  | Postprocedural or postoperative circulatory system  complication |  |  | Liveborn |  | Other specified and unspecified upper respiratory  disease |  |  | Gangrene |  |
| --- | --- | --- | --- | --- | --- | --- | --- | --- | --- | --- | --- | --- | --- |
|  |  |  |  |  |  |  | Spontaneous abortion and complications of spontaneous  abortion |  |  |  |  | Aortic; peripheral; and  visceral artery aneurysms |  |
|  |  |  |  |  |  |  |  |  |  |  |  | Aortic and peripheral arterial embolism or  thrombosis |  |
|  | Sequela of hemorrhagic cerebrovascular disease |  |  |  |  |  |  |  |  |  |  |  |  |
|  |  |  |  | Postprocedural or postoperative  digestive system complication |  |  |  |  |  |  |  |  |  |
|  |  |  |  |  |  |  | Early or  threatened labor |  |  |  |  | Disorders of teeth and gingiva |  |
|  |  |  |  |  |  |  |  |  | Chronic obstructive pulmonary disease and bronchiecta  sis |  |  |  |  |
|  | Occlusion or  stenosis of precerebral or cerebral arteries without  infarction |  |  |  |  |  | Previous C- section |  |  |  |  | Otitis media |  |
|  |  |  |  | Postprocedural or postoperative endocrine or metabolic complication |  |  |  |  |  |  |  |  |  |
|  |  |  |  |  |  |  |  |  |  |  |  | Diseases of middle ear  and mastoid (except otitis media) |  |
|  |  |  |  |  |  |  | Maternal care  for abnormality of pelvic organs |  |  |  |  |  |  |
|  |  |  |  |  |  |  |  |  |  |  |  | Diseases of inner ear and  related conditions |  |
|  |  |  |  |  |  |  |  |  | Asthma |  |  |  |  |
|  |  |  |  |  |  |  | Maternal care related to disorders of the placenta and  placental implantation |  |  |  |  |  |  |
|  | Other and ill- defined cerebrovascular  disease |  |  | Implant, device  or graft related encounter |  |  |  |  | Pleurisy, pleural effusion and  pulmonary collapse |  |  |  |  |
|  |  |  |  |  |  |  |  |  |  |  |  | Other specified and unspecified disorders of  the ear |  |
|  |  |  |  | Postprocedural or postoperative genitourinary system  complication |  |  |  |  |  |  |  |  |  |
|  |  |  |  |  |  |  |  |  |  |  |  | Fluid and electrolyte  disorders |  |
|  | Sequela of cerebral infarction and other cerebrovascular  disease |  |  |  |  |  |  |  |  |  |  |  |  |
|  |  |  |  |  |  |  | Hypertension and hypertensive- related conditions complicating pregnancy; childbirth; and  the puerperium |  |  |  |  |  |  |
|  |  |  |  |  |  |  |  |  |  |  |  | Cornea and external disease |  |
|  |  |  |  |  |  |  |  |  | Respiratory failure;  insufficien cy; arrest |  |  |  |  |
|  |  |  |  | Complication of cardiovascular device, implant or graft,  initial encounter |  |  |  |  |  |  |  | Retinal and vitreous  conditions |  |
|  | Parkinson`s disease |  |  |  |  |  |  |  |  |  |  | Neuro-ophthalmology |  |
|  |  |  |  |  |  |  |  |  | Lung disease due  to external agents |  |  |  |  |
|  |  |  |  |  |  |  |  |  |  |  |  | Oculofacial plastics and  orbital conditions |  |
|  | Multiple sclerosis |  |  |  |  |  |  |  |  |  |  |  |  |
|  |  |  |  | Complication of genitourinary device, implant or graft,  initial encounter |  |  |  |  |  |  |  | Blindness and vision  defects |  |
|  | Other specified  hereditary and degenerative nervous  system conditions |  |  |  |  |  | Prolonged pregnancy |  | Pneumotho rax |  |  |  |  |
|  |  |  |  |  |  |  |  |  |  |  |  | Encounter for administrative purposes |  |
|  |  |  |  |  |  |  | Complications  specified during childbirth |  | Mediastina  l disorders |  |  |  |  |
|  |  |  |  |  |  |  |  |  |  |  |  | Encounter for observation and examination for conditions  ruled out (excludes infectious disease, neoplasm, mental disorders) |  |
|  |  |  |  | Complication of  internal orthopedic device or implant,  initial encounter |  |  |  |  | Other specified and unspecified lower  respiratory disease |  |  |  |  |
|  |  |  |  |  |  |  | Complications specified during  the puerperium |  |  |  |  |  |  |
|  | Paralysis (other than cerebral  palsy) |  |  |  |  |  |  |  |  |  |  |  |  |
|  |  |  |  |  |  |  | Other specified complications in  pregnancy |  |  |  |  |  |  |
|  | Epilepsy; convulsions |  |  |  |  |  |  |  |  |  |  |  |  |
|  |  |  |  | Complication of transplanted organs or tissue, initial  encounter |  |  |  |  |  |  |  |  |  |
|  | Headache; including migraine |  |  |  |  |  | Uncomplicated pregnancy, delivery or puerperium |  | Respiratory signs and symptoms |  |  | Other aftercare encounter |  |
|  |  |  |  |  |  |  |  |  |  |  |  | Personal/family history  of disease |  |
|  | Neurocognitive  disorders |  |  |  |  |  |  |  |  | |  | Other specified status |  |
|  |  |  |  | Complication of other surgical or medical care, injury,  initial encounter |  |  |  |  |  |  |  |  |  |
|  |  |  |  |  |  |  | |  | | |  | Other specified and unspecified diseases of bladder and  urethra |  |
|  | Transient cerebral  ischemia |  |  |  |  |  |  |  |  |  |  |  |  |
|  | Coma; stupor; and brain  damage |  |  |  |  |  |  |  |  |  |  |  |  |
|  |  |  |  |  |  |  |  |  |  |  |  | Foodborne intoxications |  |
|  |  |  |  | Complication of cardiovascular device, implant or graft,  subsequent encounter |  |  |  |  |  |  |  |  |  |
|  |  |  |  |  |  |  |  |  |  |  |  | Amputation of a limb,  initial encounter |  |
|  | Polyneuropathie  s |  |  |  |  |  |  |  |  |  |  |  |  |
|  |  |  |  |  |  |  |  |  |  |  |  | Effect of foreign body entering opening, initial encounter |  |
|  | Sleep wake  disorders |  |  |  |  |  |  |  |  |  |  |  |  |
|  | Nerve and nerve |  |  | | |  |  |  |  |  |  | Effect of other external |  |
|  |  |  |  |  |  |  |  |  |  |  |  |  |  |

|  | root disorders |  |  | Postprocedural or postoperative musculoskeletal system  complication |  |  |  |  | causes, initial encounter |  |
| --- | --- | --- | --- | --- | --- | --- | --- | --- | --- | --- |
|  | Myopathies |  |  |  |  |  |  |  | Poisoning by drugs, |  |
|  |  |  |  |  |  |  |  |  | initial encounter |  |
|  | Nervous system  pain and pain syndromes |  |  |  |  |  |  |  |  |  |
|  |  |  |  |  |  |  |  |  | Adverse effects of drugs and medicaments, initial  encounter |  |
|  |  |  |  | Postprocedural or postoperative nervous system  complication |  |  |  |  |  |  |
|  | Other specified  nervous system disorders |  |  |  |  |  |  |  |  |  |
|  |  |  |  |  |  |  |  |  | Drug induced or toxic related condition |  |
|  | Nervous system signs and  symptoms |  |  |  |  |  |  |  | Allergic reactions |  |
|  |  |  |  | Postprocedural or postoperative respiratory  system complication |  |  |  |  |  |  |
|  |  |  |  |  |  |  |  |  | Maltreatment/abuse |  |
|  |  |  |  |  |  |  |  |  | Complication of internal orthopedic device or implant,  subsequent encounter |  |
|  | | |  |  |  |  |  |  |  |  |
|  |  |  |  | Postprocedural or postoperative  skin complication |  |  |  |  |  |  |
|  |  |  |  |  |  |  |  |  | Complication of other surgical or medical care, injury,  subsequent encounter |  |
|  |  |  |  | | |  |  |  |  |  |
|  |  |  |  |  |  |  |  |  | Schizophrenia spectrum |  |
|  |  |  |  |  |  |  |  |  | and other psychotic |  |
|  |  |  |  |  |  |  |  |  | disorders |  |
|  |  |  |  |  |  |  |  |  | Depressive disorders |  |
|  |  |  |  |  |  |  |  |  | Bipolar and related |  |
|  |  |  |  |  |  |  |  |  | disorders |  |
|  |  |  |  |  |  |  |  |  | Other specified and |  |
|  |  |  |  |  |  |  |  |  | unspecified mood |  |
|  |  |  |  |  |  |  |  |  | disorders |  |
|  |  |  |  |  |  |  |  |  | Anxiety and fear-related |  |
|  |  |  |  |  |  |  |  |  | disorders |  |
|  |  |  |  |  |  |  |  |  | Trauma- and stressor- |  |
|  |  |  |  |  |  |  |  |  | related disorders |  |
|  |  |  |  |  |  |  |  |  | Disruptive, impulse- |  |
|  |  |  |  |  |  |  |  |  | control and conduct |  |
|  |  |  |  |  |  |  |  |  | disorders |  |
|  |  |  |  |  |  |  |  |  | Personality disorders |  |
|  |  |  |  |  |  |  |  |  | Suicidal |  |
|  |  |  |  |  |  |  |  |  | ideation/attempt/intention |  |
|  |  |  |  |  |  |  |  |  | al self-harm |  |
|  |  |  |  |  |  |  |  |  | Miscellaneous mental |  |
|  |  |  |  |  |  |  |  |  | and behavioral |  |
|  |  |  |  |  |  |  |  |  | disorders/conditions |  |
|  |  |  |  |  |  |  |  |  | Alcohol-related disorders |  |
|  |  |  |  |  |  |  |  |  | Opioid-related disorders |  |
|  |  |  |  |  |  |  |  |  | Cannabis-related |  |
|  |  |  |  |  |  |  |  |  | disorders |  |

|  |  |  |  |  | Other specified bone disease and  musculoskeletal deformities |  |
| --- | --- | --- | --- | --- | --- | --- |
|  |  |  |  |  | Disorders of jaw |  |
|  |  |  |  |  | Sinusitis |  |
|  |  |  |  |  | Other specified  inflammatory condition of skin |  |
|  |  |  |  |  | Pressure ulcer of skin |  |
|  |  |  |  |  | Non-pressure ulcer of skin |  |
|  |  |  |  |  | Syncope |  |
|  |  |  |  |  | Fever |  |
|  |  |  |  |  | Shock |  |
|  |  |  |  |  | Symptoms of mental and  substance use conditions |  |
|  |  |  |  |  | Skin/Subcutaneous signs and symptoms |  |
|  |  |  |  |  | General sensation/perception  signs and symptoms |  |
|  |  |  |  |  | Other general signs and symptoms |  |
|  |  |  |  |  | Abnormal findings  without diagnosis |  |
|  |  |  |  |  | Sedative-related disorders |  |
|  |  |  |  |  | Stimulant-related disorders |  |
|  |  |  |  |  | Other specified substance-related disorders. |  |

Clinical diagnoses were aggregated using the Clinical Classifications Software Refined (CCSR). As a component of the Healthcare Cost and Utilization Project (HCUP), the CCSR groups over 700,000 ICD-10-CM codes into over 530 clinical diagnostic categories.

# Supplemental Table 4: ICD 10, HCPCS/CPT Codes Used In Analyses

| **Procedure Codes** | | |
| --- | --- | --- |
| **Procedure** | **ICD-10-PCS** | **HCPCS/CPT** |
| **Echo 2D Complete** | B24x | 93306 |
| **Echo 2D Stress** | -- | 93351 |
| **Stress Test Exercise** | -- | 93017 |
| **Stress Test Nuclear Exercise** | C21x | 78452 |
| **Diagnostic Coronary Angiograms** | B210x, B211x, B31x | 93451 - 93461 |
| **Coronary Angiograms with Therapeutic or Interventional Procedure** | 027x | 92920, 92921, 92924, 92925, 92928,  92929, 92933, 92934, 92937, 92938,  92941, 92943, 92944, 92973 |
| **CT Coronary Angiogram** | B22x, B32x | 75571 - 75574 |
| **Coronary Artery Bypass Graft** | B20x, B212x, B213x, 021x | 33510 - 33536 |
| **Thrombolysis** | 3E0xx17 | 92677 |
| **Cardiology Consult** | -- | 99254 |

The ICD-10 Revision Procedure Coding System (ICD-10-PCS), Current Procedural Terminology (CPT®) codes, and Healthcare Common Procedure Coding System (HCPCS) were utilized to identify cardiovascular diagnostic and therapeutic procedures

**Supplemental Table 5: Baseline Characteristics: T2MI vs. Dual Diagnosis (T2MI & T1MI)**

| Type 2 MI (no Type 1 Diagnosis) and Dual Patients (Co-Occurrence of Type 1 and 2 Diagnosis) | Type 2 N=18,606 | | Dual DX N=21,202 | | p-value |
| --- | --- | --- | --- | --- | --- |
|  | N | % | N | % |  |
| Age |  |  |  |  | <0.001 |
| Under 65 | 2,638 | 14.18 | 3,144 | 14.83 |  |
| 65-69 | 2,087 | 11.22 | 2,377 | 11.21 |  |
| 70-74 | 2,751 | 14.79 | 3,397 | 16.02 |  |
| 75-79 | 3,030 | 16.29 | 3,471 | 16.37 |  |
| 80-84 | 2,853 | 15.33 | 3,347 | 15.79 |  |
| 85+ | 5,247 | 28.20 | 5,466 | 25.78 |  |
| Gender |  |  |  |  | 0.003 |
| Female | 9,346 | 50.23 | 10,972 | 51.75 |  |
| Male | 9,260 | 49.77 | 10,230 | 48.25 |  |
| Race |  |  |  |  | <0.001 |
| Asian | 455 | 2.45 | 498 | 2.35 |  |
| Black | 2,980 | 16.02 | 3,103 | 14.64 |  |
| Hispanic | 1,710 | 9.19 | 2,237 | 10.55 |  |
| White | 13,461 | 72.35 | 15,364 | 72.46 |  |
| U.S. Region |  |  |  |  | <0.001 |
| Midwest | 4,015 | 21.58 | 4,249 | 20.04 |  |
| Northeast | 3,779 | 20.31 | 3,903 | 18.41 |  |
| South | 7,190 | 38.64 | 8,995 | 42.43 |  |
| West | 3,622 | 19.47 | 4,055 | 19.13 |  |
| Comorbidities |  |  |  |  |  |
| Atrial Fibrillation, Flutter, PSVT, VT | 10,370 | 55.73 | 10,953 | 51.66 | <0.001 |
| Chronic Ischemic Heart Disease | 10,775 | 57.91 | 14,046 | 66.25 | <0.001 |
| Hyperlipidemia | 14,671 | 78.85 | 17,162 | 80.95 | <0.001 |
| Smoking | 4,042 | 21.72 | 4,744 | 22.38 | 0.121 |
| Peripheral Vascular Disease | 3,920 | 21.07 | 4,571 | 21.56 | 0.238 |
| CVA, TIA, Carotid Stenosis, Intracranial Atherosclerotic Disease | 6,488 | 34.87 | 7,297 | 34.42 | 0.348 |
| Pulmonary Embolism/Venous Embolism or Thrombosis | 2,870 | 15.43 | 2,994 | 14.12 | 0.001 |
| Diabetes (1 & 2) | 9,549 | 51.32 | 10,857 | 51.21 | 0.827 |
| COPD & Asthma | 8,716 | 46.85 | 9,563 | 45.10 | 0.001 |
| Anemia and other coagulation disorders | 9,724 | 52.26 | 10,842 | 51.14 | 0.026 |
| CHF and Exacerbation | 12,005 | 64.52 | 13,732 | 64.77 | 0.617 |
| Liver Diseases | 3,340 | 17.95 | 3,426 | 16.16 | <0.001 |
| Kidney Diseases | 13,028 | 70.02 | 14,068 | 66.35 | <0.001 |
| Hypertension & Crisis | 17,484 | 93.97 | 20,023 | 94.44 | 0.048 |
| Gastrointestinal Diseases | 3,247 | 17.45 | 4,044 | 19.07 | <0.001 |
| Abbreviations: CVA = cerebrovascular accident. CHF = congestive heart failure. COPD = chronic obstructive pulmonary disease. MI = myocardial infarction; T1MI = type 1 MI; T2MI = type 2 MI. Q1= quartile 1. Q3 = quartile 3. SD = standard deviation. TIA = transient ischemic attack. * = Within 6 months before index. | | | | | |

**Supplemental Table 6: Distribution of Patients Based on Unit Type**

|  | Type 1 N=121,738 | | Type 2 N=18,606 | |
| --- | --- | --- | --- | --- |
|  | Total | | Total | |
| Ward | N | % | N | % |
| Med--Surg Floor | 27,306 | 22.43 | 5,827 | 31.32 |
| Subacute Care | 323 | 0.27 | 126 | 0.68 |
| Intensive Care | 62,518 | 51.35 | 9,593 | 51.56 |
| Coronary/Cardiology Specific Unit | 31,471 | 25.85 | 3,033 | 16.3 |
| Other/Unknown | 120 | 0.10 | 27 | 0.14 |
| Distribution of wards are different between T1 and T2 using chi-square test with significance < 0.001  Cardio Consults during index admission  If multiple wards were detected during index admission, most advanced was chosen (Med-Surg < Subacute < Intensive < Coronary/Cardiology) | | | | |

**Supplemental Table 7: Home Medications and Diagnostic or Therapeutic Procedures 6 Months Before Admit**

|  | MI Subtype | | | |  |
| --- | --- | --- | --- | --- | --- |
|  | T1MI | | T2MI | |  |
|  | N | % | N | % | P-Value |
| All | 121,738 |  | 18,606 |  |  |
| **Home Medications*** |  |  |  |  |  |
| ACEi or ARB | 52,048 | 42.75 | 7,727 | 41.53 | 0.002 |
| Aspirin | 1,461 | 1.20 | 160 | 0.86 | <0.001 |
| Beta Blockers | 46,860 | 38.49 | 8,291 | 44.56 | <0.001 |
| P2Y12 Inhibitors | 17,395 | 14.29 | 2,077 | 11.16 | <0.001 |
| Sacubitril/Valsartan | 2,597 | 2.13 | 299 | 1.61 | <0.001 |
| PCSK9 or Ezetimibe | 2,438 | 2.00 | 278 | 1.49 | <0.001 |
| GLP1-Agonist or SGLT2-i | 16,982 | 13.95 | 2,186 | 11.75 | <0.001 |
| Statins | 53,323 | 43.80 | 8,397 | 45.13 | 0.001 |
| **Pre-Admit Procedures*** |  |  |  |  |  |
| ECHO Complete | 34,562 | 28.39 | 7,637 | 41.05 | <0.001 |
| Echo Stress | 785 | 0.64 | 89 | 0.48 | 0.007 |
| Exercise Stress | 5,708 | 4.69 | 733 | 3.94 | <0.001 |
| Nuclear Stress | 11,700 | 9.61 | 1,620 | 8.71 | <0.001 |
| Invasive Angiogram | 9,013 | 7.40 | 1,331 | 7.15 | 0.224 |
| CT Coronary Angiogram | 392 | 0.32 | 112 | 0.60 | <0.001 |
| PCI | 3,970 | 3.26 | 332 | 1.78 | <0.001 |
| CABG | 1,159 | 0.95 | 159 | 0.85 | 0.199 |
| Abbreviations: ACEi = angiotensin converting enzyme inhibitor. ARB= angiotensin receptor blocker. CT= Computed Tomography. ECHO = echocardiogram. GLP = glucagon like peptide-1. MI = myocardial infarction; T1MI = type 1 MI; T2MI = type 2 MI. P2Y12 = purigenic receptor inhibitor. PCSK9 = paraprotein convertase subtilisin/kexin type 9. PSVT = paroxysmal supraventricular tachycardia. PCI = percutaneous coronary intervention. Q1= quartile 1. Q3 = quartile 3. SD = standard deviation. SGLT2-i = sodium glucose co-transporter 2 inhibitor. TIA = transient ischemic attack. VT = ventricular tachycardia. * = Within 6 months before index | | | | | |

# Supplemental Table 8: Sensitivity Analysis of Procedure Rates Among Patients with Primary

# Diagnosis of T2MI Without Concurrent Secondary Diagnosis.

| Procedures During Index Admit or Within 6 Months of Discharge | Type 1 (N*=99,593) | | Type 2 Primary Diagnosis (N*=641) | | Odds of Procedure/Intervention in T2 vs. T1 OR [95% CI] | |
| --- | --- | --- | --- | --- | --- | --- |
|  | N | % | N | % | Unadjusted | Adjusted^+^ |
| Echo Complete | 82,551 | 82.89% | 481 | 75.04% | 0.62 [0.52, 0.74]* | 0.55 [0.46, 0.67]* |
| Echo Stress | 477 | 0.48% | NR | -- | 0.65 [0.16, 2.61] | 0.57 [0.14, 2.30] |
| Stress Exercise | 3,245 | 3.26% | 24 | 3.74% | 1.15 [0.77, 1.74] | 1.10 [0.72, 1.66] |
| Stress Nuclear | 9,388 | 9.43% | 109 | 17.00% | 1.97 [1.60, 2.42]* | 1.56 [1.26, 1.93]* |
| Angiogram | 76,327 | 76.64% | 181 | 28.24% | 0.12 [0.10, 0.14]* | 0.12 [0.09, 0.14]* |
| CT Angiogram | 1,132 | 1.14% | 11 | 1.72% | 1.52 [0.83, 2.76] | 1.10 [0.60, 2.01] |
| PCI | 49,470 | 49.67% | 38 | 5.93% | 0.06 [0.05, 0.09]* | 0.08 [0.06, 0.12]* |
| CABG | 18,292 | 18.37% | 32 | 4.99% | 0.23 [0.16, 0.33]* | 0.25 [0.17, 0.37]* |
| Cardiologist Consult/Visit | 86,275 | 86.63% | 591 | 92.20% | 1.82 [1.37, 2.44]* | 1.07 [0.78, 1.46] |
| CABG = coronary artery bypass graft. CT = computed tomography. ECHO = echocardiogram. T1MI = type 1 MI; T2MI = type 2 MI. PCI = percutaneous coronary intervention.  * Patients who expired in hospital, discharged from hospital, or did not have 6 months of continuous enrollment were excluded for this table.  + Adjusted for demographics, comorbidities, primary diagnosis, and pre-admit testing  NR: Not Reportable due to small cell size (between 1 and 10) | | | | | | |

**Supplemental Table 9: Proportion Of Patients Who Underwent PCI/CABG After Coronary Computed Tomography Angiography (CCTA) or Invasive Angiogram.**

|  | N | PCI | % | CABG | % | PCI or CABG | % |
| --- | --- | --- | --- | --- | --- | --- | --- |
| T1MI | 76,385 | 49,200 | 64.41% | 18,004 | 23.57% | 60,596 | 79.33% |
| T2MI | 1,740 | 252 | 14.48% | 201 | 11.55% | 414 | 23.79% |
| N = Total number of patients who underwent CCTA or invasive angiography prior to therapeutic intervention.  CABG = coronary artery bypass graft. T1MI = type 1 MI; T2MI = type 2 MI. PCI = percutaneous coronary intervention.  Procedures during index or within 6 months post discharge  *Proportions of all procedures between T1 and T2 are different using chi-square tests with significance less than 0.001.  Patients who expired in hospital, discharged to hospice, or did not have 6 months of continuous enrollment were excluded for this table. | | | | | | | |
